# Two major QTL for Cadmium accumulation in oat (*Avena sativa* L.) grains

**DOI:** 10.1007/s00122-026-05427-y

**Published:** 2026-11-11

**Authors:** Fluturë Novakazi, Pernilla Vallenback, Alf Ceplitis

**Affiliations:** 1 Department of Plant Pathology and Sustainable Crop Protection, Faculty for Agriculture, Civil and Environmental Engineering, University of Rostock, Rostock, Germany; 2 Lantmännen Lantbruk, Svalöv, Sweden; 3 Swedish University of Agricultural Sciences, Southern Swedish Forest Research Centre, Alnarp, Sweden

## Abstract

**Abstract:**

Cadmium (Cd) is a heavy-metal that accumulates in the environment, in cereal grains and ultimately in humans posing significant health risks. With the increased demand on plant-based products, the production of oats for food products has increased as well, and with it the risk of uptake of Cd. However, limited research exists on the genetic control of Cd accumulation in oat grains. This study evaluated a panel of 620 Nordic spring oat breeding lines in field trials in Sweden during 2015–2021. GWAS was conducted for 596 lines for Cd grain content, thousand kernel weight (tkw), and hectolitre weight (hlw). Fifteen and six significant marker trait associations were identified for tkw and hlw, respectively. Some of which corresponded to previously reported QTL and some of which represent putatively new loci. Two new major QTL were identified associated with Cd grain accumulation located on chromosomes 3D (432.54 Mbp) and 4A (381.23 Mbp), explaining 34.69% and 27.92% of the phenotypic variation, respectively.

***Key message*:**

**A total of 596 oat breeding lines were analysed for Cadmium accumulation in the grains. Genome-wide association studies identified two major QTL as well as strong candidate genes.**

**Supplementary Information:**

The online version contains supplementary material available at https://doi.org/10.1007/s00122-026-05427-y.

## Introduction

Oats (*Avena sativa*) rank sixth in worldwide cereal production after maize, rice, wheat, barley, and sorghum, with the European Union being the biggest producer (FAO 2023). At the end of the 1960s the world oat production reached its peak with almost 55 million tons produced annually, but has since continually decreased and stabilised at about 22 million tons in the last ten years (FAO 2023). The decline in oat production is attributed to the mechanisation in agriculture and, as a consequence, the decreased demand of oats as feed for horses, which until then were used in land cultivation.

Today, most of the production is still used as animal feed, but with the recognition of its health benefits in the 1990s, oat production for human consumption is increasing (Ma et al. 2021). Oats have the highest oil content (6–12%) and protein content (up to 20%) among small grain cereals and are high in β-glucans and the essential fatty acid linoleic, which are known to reduce blood cholesterol and improve heart health (EFSA Panel on Dietetic Products, Nutrition and Allergies (NDA) 2010; Ma et al. 2021). Additionally, it is rich in antioxidants, alpha-tocotrienol, alpha-tocopherol and avenanthramides, minerals and vitamins (Peterson 2001). Oats are suitable for patients with type 2 diabetes, because it has a low glycaemic index (Tapola et al. 2005). In recent years, the unique characteristics of oats have also been discovered by the cosmetics industry. With the increased demand of consumers for non-dairy and locally grown products, a market for oat-based food products has developed and the range of products is consistently growing.

Cadmium (Cd) is a highly toxic non-essential heavy metal, which is very persistent and therefore considered an environmental contaminant (Genchi et al. 2020). It can remain in the environment for several decades and accumulates in the soil. Cd is mainly released through industrial activities like mining, refining and production of plastic (Genchi et al. 2020). However, it also enters the soil through fertilizers and plant protection products that contain Cd, sewage sludge and manure (Chunhabundit 2016).

Plants take up Cd from the soil through the root system. It is then translocated to other plant parts and accumulates in leaves and storage organs (Abedi and Mojiri 2020; Hu et al. 2022). High amounts of Cd negatively impact germination, root elongation, and overall growth in plants. It interferes with respiration and photosynthesis by affecting for example chlorophyll production (Rizwan et al. 2016; Abedi and Mojiri 2020). Additionally, increased Cd uptake has been linked to an elevated production of reactive oxygen species (ROS) and an overall imbalance of nutrient uptake (Rizwan et al. 2016; Çatav et al. 2020; Abedi and Mojiri 2020). Ultimately, it leads to decreased biomass production and reduced yield. In cereal crops, like rice, wheat and oat, accumulation of Cd in the grains is especially problematic, since humans consume cereal products in large quantities. In humans, Cd accumulates mainly in the kidney, leading to kidney damage or even failure. Furthermore, high Cd accumulation in humans has been linked to bone demineralisation, as well as to lung, bladder and breast cancer (Chunhabundit 2016). The EU has limited the maximum Cd in cereal products to 0.05 to 0.2 mg/kg wet weight and to 0.04 mg/kg for cereals intended for baby food production (Commission Regulation [EU] 2021). However, with the increasing demand of plant-based products, an increased intake of Cd through food can be expected.

In Europe, soils contain on average 0.2 mg/ kg of Cd, with regional variations among, but also within, countries (Ballabio et al. 2024). However, in many regions average Cd soil content exceeds 0.2 mg/ kg, with many local soils reaching Cd values of > 1 mg/ kg (Ballabio et al. 2024). These high Cd soils often overlap with regions used for cereal production in Europe, increasing the risk of food products containing high amounts of Cd and its effects on human health (Eurostat 2025). Especially oats are often used for baby food production, putting this vulnerable group at an even higher risk for Cd related health issues.

Generally, the higher the total amount of Cd in the soil, the more the plant will take up. However, the plant available Cd is affected by the solubility of soil Cd, pH, soil type, clay and organic matter content, soil fertility and weather conditions, like precipitation during the growth season, as well as the interplay of these factors (Eriksson et al. 1990, 1996; Eriksson 1990; He and Singh 1993; Cieśliński et al. 1996; Liu et al. 2015). Additionally, genetic differences in Cd uptake and accumulation between and within species have been reported for several crop species (Florijn and Van Beusichem 1993; Yang et al. 1995; Cieśliński et al. 1996; Welch and Norvell 1999; Arao et al. 2003; Eurola et al. 2003; Arao and Ishikawa 2006).

Plants take up aqueous forms of Cd present in the soil solution. Aqueous species of Cd are Cd^2+^ and complexed or chelated inorganic and organic forms (Welch and Norvell 1999). Cd^2+^ cations are absorbed either through the electrochemical potential at the root surface across the plasma membrane, through Cd specific membrane transporters or through transporters specific for other divalent cations like Fe^2+^, Mg^2+^or Zn^2+^ (Welch and Norvell 1999; Uraguchi et al. 2011). Several protein families identified in different plant species have been reported to be involved in Cd uptake by the roots. Amongst these protein families are zinc-regulated transporter (*ZRT*)/ iron-regulated transporter (*IRT*) like proteins (*ZIP*), natural resistance-associated macrophage proteins (*NRAMP*), or low-affinity cation transporters (*LCT*) (Uraguchi et al. 2011; Abedi and Mojiri 2020; Tao and Lu 2022). Cd chelates enter the root through yellow stripe 1-like (*YSL*) proteins (Curie et al. 2009). Once in the root, Cd is translocated into the shoot and leaves via the xylem. A major regulator of root-to-shoot translocation of Cd is the heavy metal ATPase (*HMA*) family, which are tonoplast-localized transporters. Overexpression of *OsHMA3* and *TaHMA3* in rice and wheat respectively, decreased the root-to-shoot translocation significantly by sequestering Cd into the vacuoles (Salt and Wagner 1993; Ueno et al. 2009; Sasaki et al. 2014; Zhang et al. 2020b). HMA proteins with major effect on Cd grain accumulation have also been reported in maize and soybean (Benitez et al. 2012; Tang et al. 2021a). Overexpression of the rice membrane protein *OsLCT2* reduced the translocation from the root to the shoot significantly by limiting the amounts of Cd loaded into the xylem (Tang et al. 2021b). In a genome-wide transcriptome study on barley plants exposed to high Cd concentration, Kintlová et al. (2021) identified a gene upregulated in root and shoot tissue, *PLANT CADMIUM RESISTANCE 2* (*HvPCR2*). Additionally, they identified four *HvPCR2* genes located in a tandem repeat arrangement on chromosome 5H. Finally, translocation from the shoot into the grain is related to phloem-mediated Cd transport. In rice, two *LCT* genes, *OsLCT1* and *OsLCT2* were reported. *OsLCT1* was found to regulate Cd translocation from the shoot to the grain. Loss of function mutants of *OsLCT1* accumulated half as much Cd in the grain as the wild type (Uraguchi et al. 2011). Overexpression of *OsLCT2* limited Cd accumulation in the shoot and the seeds (Tang et al. 2021b).

Genome-wide association studies to identify genomic regions associated with Cd uptake or accumulation were reported for several crop species, e.g. barley (Wu et al. 2015), bread wheat (Hussain et al. 2020; Safdar et al. 2020), durum wheat (AbuHammad et al. 2016), oil seed rape (Chen et al. 2018) and maize (Zhao et al. 2018). Even though the regulation of Cd uptake and accumulation in the plant is most likely controlled by several genes, major genes and QTL have been identified through QTL mapping in many crop species. Ishikawa et al. (2010) identified two major QTL located on chromosome 2 and 7S associated with increased Cd grain concentration in rice. Three QTL associated with shoot Cd concentration were identified on rice chromosomes 2, 5 and 11 (Ueno et al. 2009). Benitez et al. (2010) identified a major QTL in a soybean RIL population controlling Cd concentration in the seed, which accounted for 57–82% of the genetic variation. In durum wheat a major QTL located on chromosome 5BL was identified in a RIL population explaining 54.3% of the phenotypic variation (AbuHammad et al. 2016). Derakhshani et al. (2020) identified a major and a minor QTL in a barley mapping population on chromosomes 6H and 2H, respectively. Subsequent RNA-Seq analysis revealed 16 candidate genes related to Cd tolerance. Several genes have already been cloned. For example, Benitez et al. (2010) and Ueno et al. (2010) cloned the genes *GmHMA1* in soybean and *OsHMA3* in rice, respectively. Both genes encode P_1B_-ATPases, and were primarily expressed in the roots.

Little research has been done in regard to Cd uptake and accumulation in oat grains. Tanhuanpää et al. (2007) identified a major gene for Cd grain accumulation in oats in an F_2_ population of a cross between the oat cultivars ‘Aslak’ and ‘Salo’ (high Cd accumulator). However, the exact location of the gene could not be determined at the time. To our knowledge, no follow-up studies by the authors, no other QTL mapping nor GWAS studies have been published on the subject to date.

In the present study, a spring oat breeding population of 620 genotypes were grown in field trials in Svalöv, southern Sweden in the years 2015 to 2021. The harvested oat kernels were analysed for thousand kernel weight, hectolitre weight (test weight) and Cd content in the grain. The aim was to dissect the genetic architecture underlying Cd accumulation in oat grains and to identify its correlation with important quality traits in oats. To identify genomic regions associated with these three traits and putative candidate genes for Cd accumulation, we conducted genome-wide association studies and mapped the significant markers to the ‘Sang’ oat reference genome.

## Materials and methods

### Field experiments

The present data was collected by the Lantmännen Plant Breeding unit within their spring oat breeding program. The data set comprises in total 620 spring oat breeding lines. These lines were tested in yield trials in fields in Svalöv, Southern Sweden, between the years 2015 and 2021. In Lantmännen’s oat breeding program, the breeding lines are tested by default in an augmented design with incomplete blocks and one replication. Each year, 88 lines enter the field trials and are tested for three consecutive years. Additionally, the three check cultivars, namely Belinda, Galant and Nike, were replicated in each block. These three cultivars are included as checks in the Swedish official variety registration (VCU – Value for Cultivation and Use) and are therefore included in Lantmännen’s yield trials. ‘Galant’ is known to accumulate high amounts of Cd in the grain, but is nonetheless the most popular oat cultivar grown in Sweden. The plot size was 14 m^2^. Plants were fertilized twice with nitrogen and treated with fungicides if necessary.

### Trait assessment

After harvest, phenotypic data was recorded for hectolitre weight (hlw), thousand kernel weight (tkw) and cadmium content in the grain. Hlw and tkw were analysed with the Cgrain Value™ seed analyser (Cgrain AB, Sweden) at Lantmännen’s seed laboratory in Svalöv.

Cadmium (Cd) grain content was determined with an in-house method at Lantmännen’s lab employing a microwave assisted extraction (MAE) method followed by graphite furnace atomic absorption spectrometry (GFAAS). In short, the steps were as follows: 25 g of grain were processed into a fine powder with a hammer mill (16 000 rpm) and sieved through a 0.8 mm sieve. Of the milled sample, 0.5 g were transferred into a microwave tube, into which 7 mL of concentrated HNO_3_, 2 mL of water and 1 mL of H_2_O_2_ were added. The tubes were placed into a microwave oven, where they were slowly heated under pressure (90 bar) to 180 °C. The temperature and pressure were kept constant for 30 min. After cooling down, the samples were transferred to new tubes, filled up to 50 mL and left to settle. Subsequently, 2 mL of each sample were transferred into the GFAAS. Here, 10 µL of each sample were heated to 1500 °C. Through a lamp with a wavelength specific to Cd, the absorption level and hence the amount of Cd was measured. Finally, the furnace was heated up to 2500 °C to eliminate any remaining traces of the sample. Each sample was analysed three times. Before and after each run, standardised control samples (obtained from Wageningen University) of oat grains with defined amounts of Cd were analysed, to ensure the system is stable.

### Statistical analyses

Statistical analysis and analysis of variance (ANOVA; type III with Satterthwaite’s method) were performed in R v.4.3.1 using the *lmer* function in the package lme4 (Bates et al. 2015). Genotype and year were considered fixed effects, the remaining factors, i.e. genotype*year and block, were set as random.

The pairwise Pearson correlation coefficients between the phenotyped traits were calculated in R and visualized with the package corrplot (Wei and Simko 2017).

Best linear unbiased estimators (BLUEs) were calculated for each genotype across years with only the genotype considered as a fixed effect. The BLUEs were used as phenotypic input in the subsequent GWAS analysis.

The broad sense heritability was estimated as *H²* = V_G_/ V_P_, where V_G_ is the total genetic variance and V_P_ the total phenotypic variance. Variance components were estimated in R using the *lmer* function in lme4 setting all factors random (Bates et al. 2015). *H²* across years was calculated with the following formula:

$$\:{H}^{2}=\:\frac{{{\sigma\:}^{2}}_{G}}{{\sigma\:}^{2}_{G}+\frac{{{\sigma\:}^{2}}_{GY}}{y}+\:\frac{{{\sigma\:}^{2}}_{R}}{y}\:}$$

where *σ²*_*G*_ is the genotypic variance, *σ²*_*GY*_ the genotype *x* year variance, *σ²*_*R*_ the residual variance and y the number of years.

For the peak SNPs associated with Cd grain accumulation, a factorial ANOVA (type III) was performed to explore the significance of the SNPs and the significance of the interaction, followed by an analysis of the simple effects.

### Genotyping and population structure analyses

All breeding lines were genotyped with the 7k Illumina oat SNP chip, which is available at TraitGenetics (SGS Institute Fresenius GmbH, Germany). The chip contains 6631 SNPs, of which about 2900 SNPs overlap with the 6k Infinium SNP genotyping array (Polley et al. 2023). The SNPs were subjected to the following quality parameters: SNP call rate > 80%, and removal of monomorphic and unmapped SNPs. Genotypes appearing as duplicates were removed, as well. Subsequently, the genotypic data was imputed with Beagle v5.4 with default parameters (Browning et al. 2018).

To obtain physical positions, the SNPs from the 7k chip were mapped against the oat reference genome of oat cultivar ‘Sang’ (Kamal et al. 2022).

For population genetics analyses and GWAS 596 genotypes and 3322 SNP markers passed the quality criteria mentioned above.

In order to assess population structure, a Bayesian clustering approach implemented in STRUCTURE v.2.3.4 was employed (Pritchard et al. 2000). For each hypothetical number of sub-groups (K = 1 to 10), ten repetitions were run with a burn-in phase of 1000 and 10 000 Markov Chain Monte Carlo (MCMC) iterations. Based on the Δk method (Evanno et al. 2005) the most likely number of K was determined using STRUCTURE Harvester v0.6.94 (Earl and von Holdt 2012). STRUCTURE results were visualised with ggplot in R.

Principal Component Analysis (PCA) was performed with *smartpca* in EIGENSOFT v5 (Patterson et al. 2006) and visualised with ggplot in R. Lastly, the Van Raden kinship was calculated and visualised with the ASRgenomics package v.1.1.0 in R (Gezan et al. 2022).

Linkage disequilibrium (LD) decay was calculated in R with the function *LD.decay* from the package sommer v2.9 (Covarrubias-Pazaran 2016). The squared allele frequency correlation *r²* between markers pairs was calculated and a second-degree smoothed loess curve was fitted to the data using the base R function *loess* with span set to 0.1. The 95th percentile of the LD distribution between unlinked markers was considered as the threshold for when LD decayed. LD decay was estimated for each of the 21 oat chromosomes, for each sub-genome and across genomes.

### Genome-wide association studies

GWAS was performed with the R package GAPIT v3 (Lipka et al. 2012; Wang and Zhang 2021). Markers with a minor allele frequency < 0.02 were removed within the GWAS run, which left 2613 SNPs for GWAS. Four models, implemented in GAPIT were run for each trait, these were mixed linear model (MLM) (Zhang et al. 2010), multiple loci mixed linear model (MLMM) (Segura et al. 2012), fixed and random model circulating probability unification (FarmCPU) (Liu et al. 2016), and bayesian-information and linkage disequilibrium iteratively nested keyway (BLINK) (Huang et al. 2019). Each model was tested with the kinship matrix (K) obtained within GAPIT based on the VanRaden method (VanRaden 2008). Additionally, each model was tested with and without including the ancestry coefficients (Q matrix) from STRUCTURE or principal component covariates from GAPIT. The best model was chosen based on the least deviation from the expected *p*-values.

The significance threshold for marker trait associations (MTAs) was determined with a Bonferroni correction with α = 0.05, corresponding to a threshold of -log10(α/ nSNP) = 4.72. GWAS results were visualised with the R package CMPlot (LiLin-Yin 2015).

### Putative candidate genes

To identify putative candidate genes associated with Cd accumulation or transport a position-dependent strategy was used. The positions of the significant MTAs were compared to the oat ‘Sang’ pseudomolecule reference sequence in the GrainGenes T3/ Oat database using the genome browser (https://graingenes.org/GG3/genome_browser). Additionally, local LD was examined to determine if our significant SNPs were in LD with the proposed candidate genes. For the putative candidate genes, PCR primers were developed based on the ‘Sang’ sequence (Table S1). For the genes on chromosome 3D, primer pairs were designed based on the regions spanning from 413,053,195 to 413,057,999 bp (3D_413-f/3D_413-r) and 430,740,000 to 430,745,300 bp (3D_430-f/3D_430-r2). For the gene on chromosome 4A the primers were designed on the region spanning from 381,222,000 to 381,226,541 bp (4A_381-f/4A_381-r). This gene was on the negative strand of the ‘Sang’ genome. The targeted regions included at least 2000 bp upstream of the gene. Amplified products of five low Cd (i.e. SOA369, SOA372, Active, Hera, Welinder) and three high Cd (i.e. SOA026, SOA599, SOA608) genotypes were sequenced to identify possible polymorphisms in the gene sequence associated with the phenotype. The sequences were aligned and compared with the software BioEdit v7.2.5 (Hall 1999). Sequence information was deposited at NCBI. The accession numbers can be found in Table S2.

## Results

### Phenotypic evaluation

**Fig. 1:**
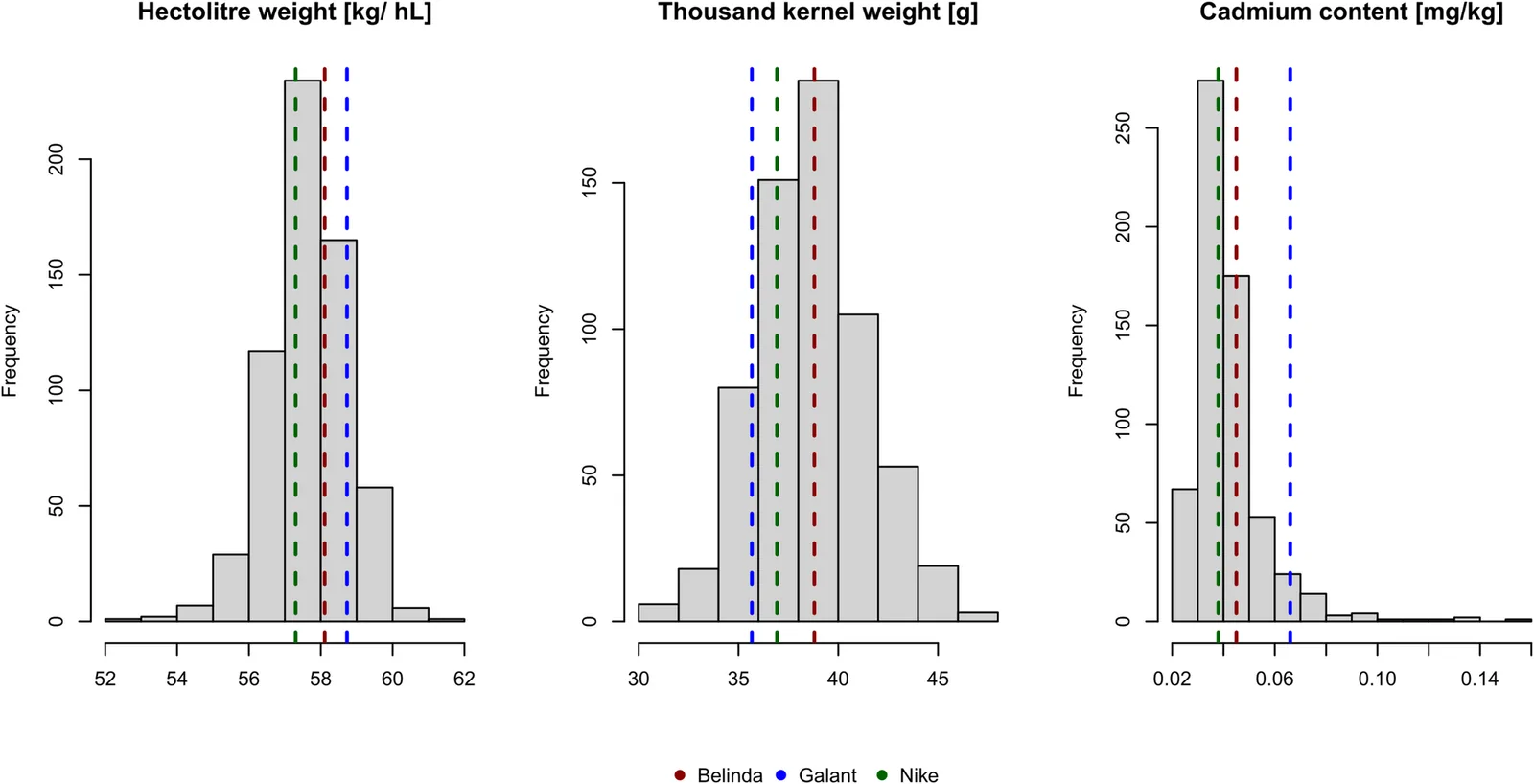
Frequency distribution of 620 spring oat breeding lines evaluated for hectolitre weight, thousand kernel weight and cadmium grain content under field conditions between 2015 to 2021 in Svalöv, Southern Sweden. The dashed lines represent the check cultivars Belinda (red), Galant (blue), and Nike (green)

Phenotypic data was analysed for hlw, tkw and Cd grain content. ANOVA showed highly significant differences among the spring oat genotypes and years for all three traits (Table 1). The values for hlw ranged from 52.3 to 61.2 kg/ hL (mean 57.6 kg/hL), the three check cultivars showed values of 58.11 kg/ hL (Belinda), 58.73 kg/ hL (Galant), and 57.30 kg/ hL (Nike) (Fig. 1; Table 2). The frequency distribution for hlw was slightly left skewed (Fig. 1). The broad sense heritability (*H²*) was 0.568 (Table 2).

**Table 1:** Analysis of variance (ANOVA) for hectolitre weight, thousand kernel weight, and cadmium grain content for 620 spring oat breeding lines evaluated under field conditions in Svalöv, Southern Sweden, from 2015 to 2021

Trait		Sum Sq	Mean Sq	DF	DenDF	F value	*p*-value
Hectolitre weight	Genotype	823.1073	1.3297	619	729.2591	1.6869	6.224E-12 ***
	Year	131.4261	21.9043	6	56.3257	27.7878	3.695E-15 ***
Thousand Kernel Weight	Genotype	5715.6497	9.2337	619	959.8870	8.7164	5.867E-189 ***
	Year	162.6008	27.1001	6	57.0310	25.5821	1.646E-14 ***
Cadmium Grain Content	Genotype	0.1967	0.0003	619	1010.4316	3.6095	1.432E-73 ***
	Year	0.0178	0.0030	6	51.3883	33.6081	3.893E-16 ***

Significance codes: ‘***’ 0.001 ‘**’ 0.01 ‘*’ 0.05

 Tkw ranged from 30.5 to 47.4 g (mean 38.6 g), with check cultivars showing values of 38.8 (Belinda), 35.7 (Galant) and 36.9 (Nike) (Fig. 1; Table 2). The broad sense heritability (*H²*) for tkw was quite high (0.896) (Table 2). The frequency distribution for cadmium grain content was right skewed, and ranged from 0.021 to 0.158 mg/kg (mean 0.042 mg/kg) (Fig. 1; Table 2). Check cultivar Galant showed with 0.066 mg/kg a comparably high cadmium grain content and six lines showed values above 0.1 mg/kg (Fig. 1). The broad sense heritability (*H²*) for cadmium grain content was 0.769, which seems quite high for a quantitative trait, however, this shows the chosen field design corrected well for random effects (Table 2)

**Table 2:** Minimum, maximum, and mean values for 620 spring oat breeding lines, evaluated for hectolitre weight, thousand kernel weight and cadmium grain content in field trials from 2015 to 2021, in Svalöv, Southern Sweden, as well as corresponding broad sense heritability (*H²*)

Trait	min	max	mean	*H²*
Hectolitre weight [kg/ hL]	52.3	61.2	57.6	0.568
Thousand Kernel Weight [g]	30.5	47.4	38.6	0.896
Cadmium Grain Content [mg/ kg]	0.021	0.158	0.042	0.769

**Fig. 2:**
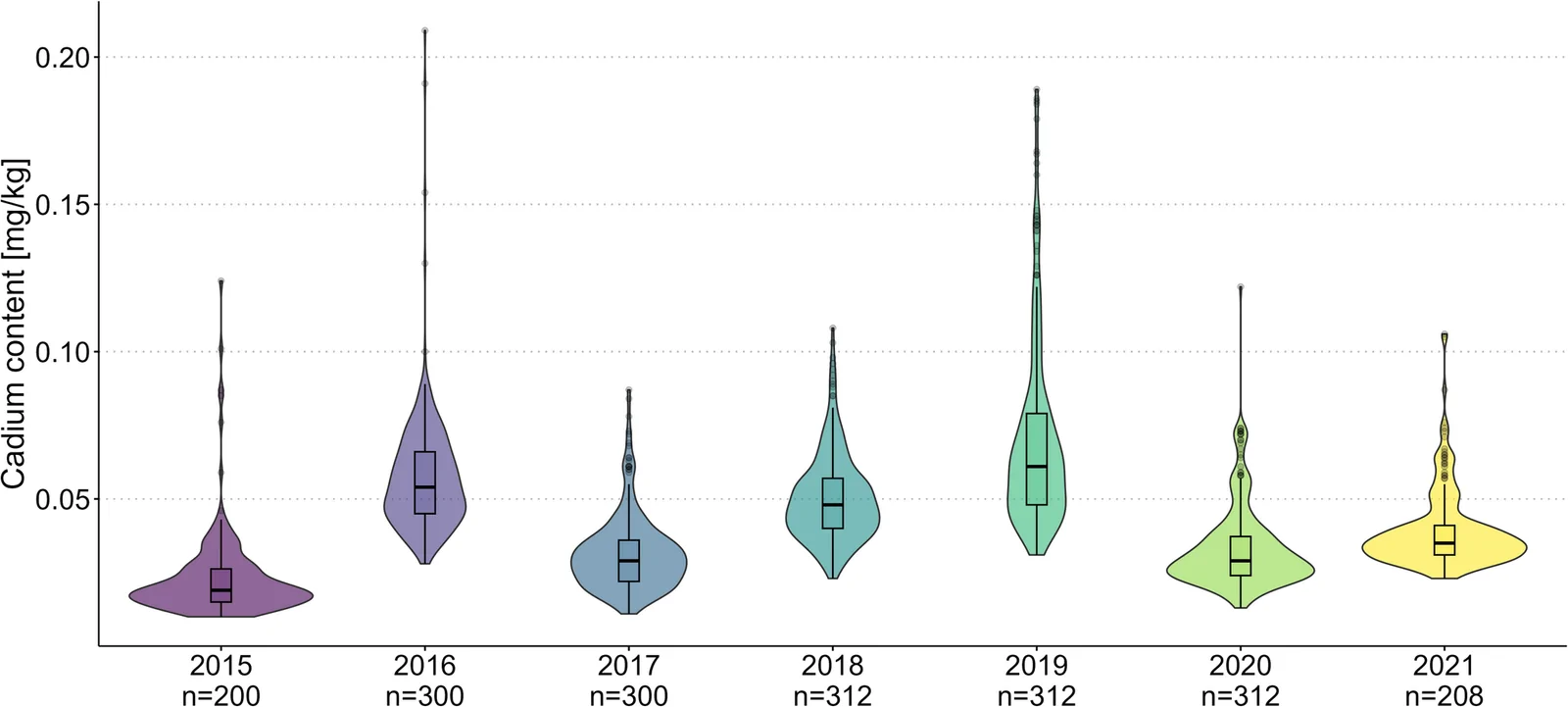
Violin plots for spring oat breeding lines grown under field conditions in Svalöv, Southern Sweden, and analysed for cadmium grain content. Box plots represent the minimum, first quartile, median, third quartile, and maximum. Numbers underneath each year represent the number of lines tested in the respective year, including the three check cultivars, ‘Belinda’, ‘Galant’, and ‘Nike’

All three traits are influenced by environmental factors, such as temperature and precipitation. This effect could be clearly observed for the period of the field trials (Fig. 2, Suppl. Figure 1). In the years 2016, 2018 and 2019 the average cadmium grain content was higher compared to the other years (Fig. 2). For tkw, the effect was the opposite, the average tkw was the lowest in 2016 and 2018, and highest in 2015 and 2017 (Suppl. Figure 1). Hlw showed the lowest values in 2016 and 2021, and the highest values in 2015, 2017 and 2018 (Suppl. Figure 1). Cd grain content and tkw showed a significant negative correlation (*r²* = -0.36), whereas Cd grain content and hlw, and tkw and hlw showed no correlation (Suppl. Figure 1).

### Population structure and linkage disequilibrium decay

**Fig. 3:**
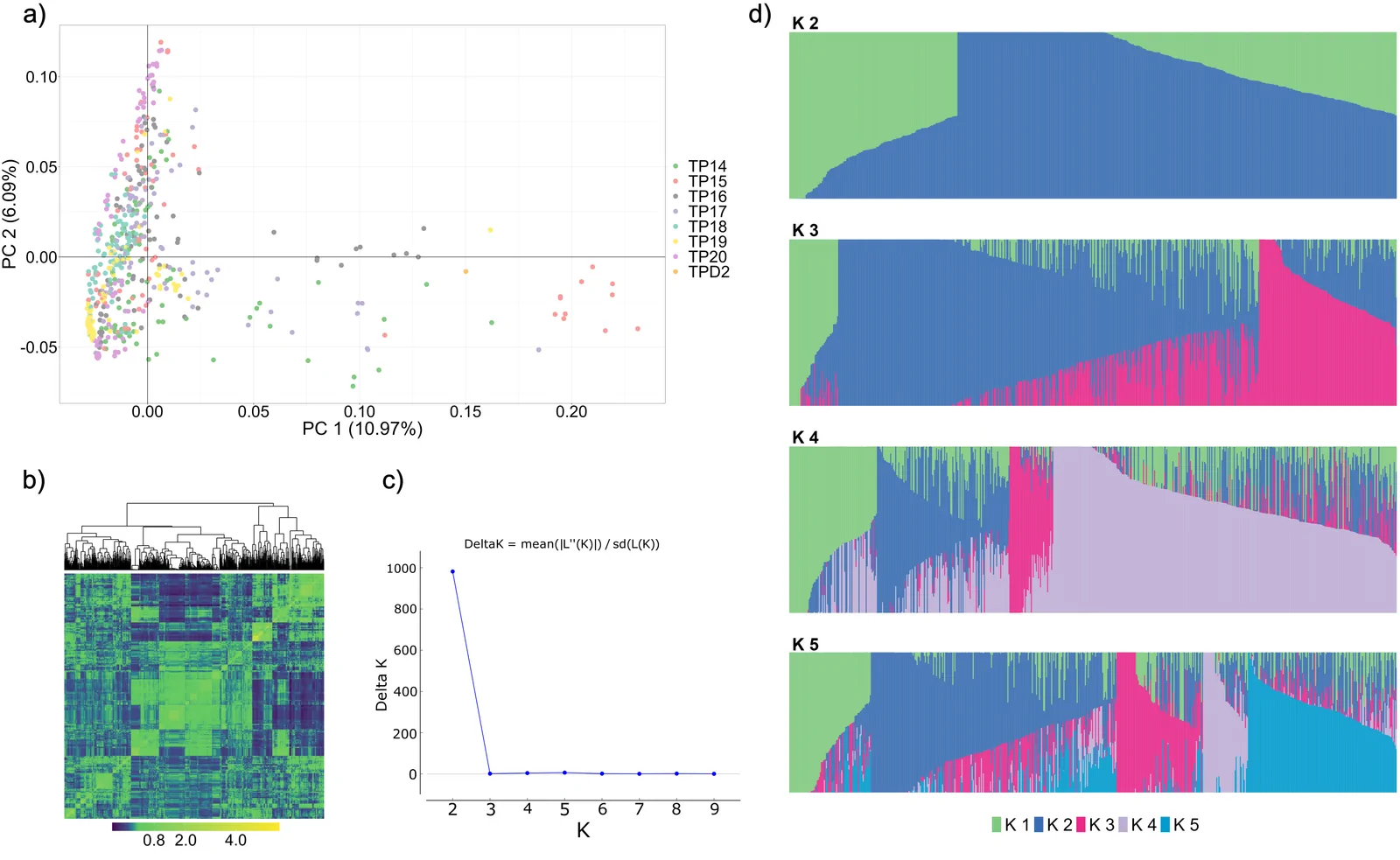
Analysis of population structure of 596 spring oat breeding lines with 3322 SNP markers. (**a**) Principal component analysis (PCA) plot of the first two principal components with breeding lines coloured according to the year they entered the field trials, (**b**) heat map of VanRaden Kinship, (**c**) delta K plot and (**d**) bar plots of ancestry analysis with STRUCTURE

PCA showed a weak population structure as most lines clustered together (Fig. 3a). The first two principal components explained 10.97% and 6.09% of the phenotypic variation, respectively. The kinship supported the weak population structure, showing only moderate levels of relatedness between the analysed breeding lines, which can be expected for a breeding population consisting of elite germplasm (Fig. 3b).

Analysis of ancestry revealed an optimal k value of 2, with a smaller peak at k = 5 (Fig. 3c). Genotypes showing ancestry coefficient < 0.80 to one subpopulation were considered admixed. For k = 2, 49 lines belonged to subpopulation K1, 243 to K2 and 304 showed admixed ancestry. For k = 5, 442 lines were admixed, 31 belonged to K1, 70 to K2, 22 to K3, 17 to K4, and 14 to K5 (Fig. 3d).

LD decay varied across genomes and chromosomes (Table 3, Suppl. Figure 2). For sub-genome A, LD decayed at 28.7 Mbp, for sub-genome C at 32.92 Mbp, and for sub-genome D at 35.71 Mbp. Across chromosomes LD decay varied between 7.08 Mbp (7 C) and 332.92 Mbp (1 C).

**Table 3:** Linkage disequilibrium (LD) decay of a population of 596 Nordic spring oat breeding lines calculated for each sub-genome and respective chromosomes

	Mbp	r2	# SNPs
**Genome A**	28.70	0.0921	1067
1 A	28.41	0.1282	198
2 A	42.77	0.0824	112
3 A	13.11	0.0714	123
4 A	26.70	0.0826	242
5 A	126.68	0.0759	152
6 A	24.95	0.1015	95
7 A	28.63	0.1030	145
**Genome C**	32.92	0.0894	914
1 C	332.90	0.0834	48
2 C	14.58	0.0848	97
3 C	16.35	0.0885	103
4 C	38.47	0.1006	222
5 C	16.76	0.0798	156
6 C	21.20	0.0949	187
7 C	7.08	0.0937	101
**Genome D**	35.71	0.0907	1308
1D	34.92	0.0937	339
2D	27.94	0.0780	201
3D	13.81	0.0768	76
4D	27.44	0.0906	97
5D	12.52	0.1087	182
6D	195.71	0.0668	173
7D	38.41	0.1199	240
**Across genomes**	31.601	0.0950	3289

### Model selection and marker trait associations

Four models were tested in GAPIT: MLM, MLMM, FarmCPU and Blink. Each model was additionally tested with PC = 0 and PC = 5 as covariates obtained from GAPIT and with the Q matrix obtained from STRUCTURE assuming k = 5 (Q5). The best model for hlw was FarmCPU + Q5, for tkw BLINK + Q5, and the best model for Cd grain content was MLMM + Q5 (Suppl. Figure 3).

**Fig. 4:**
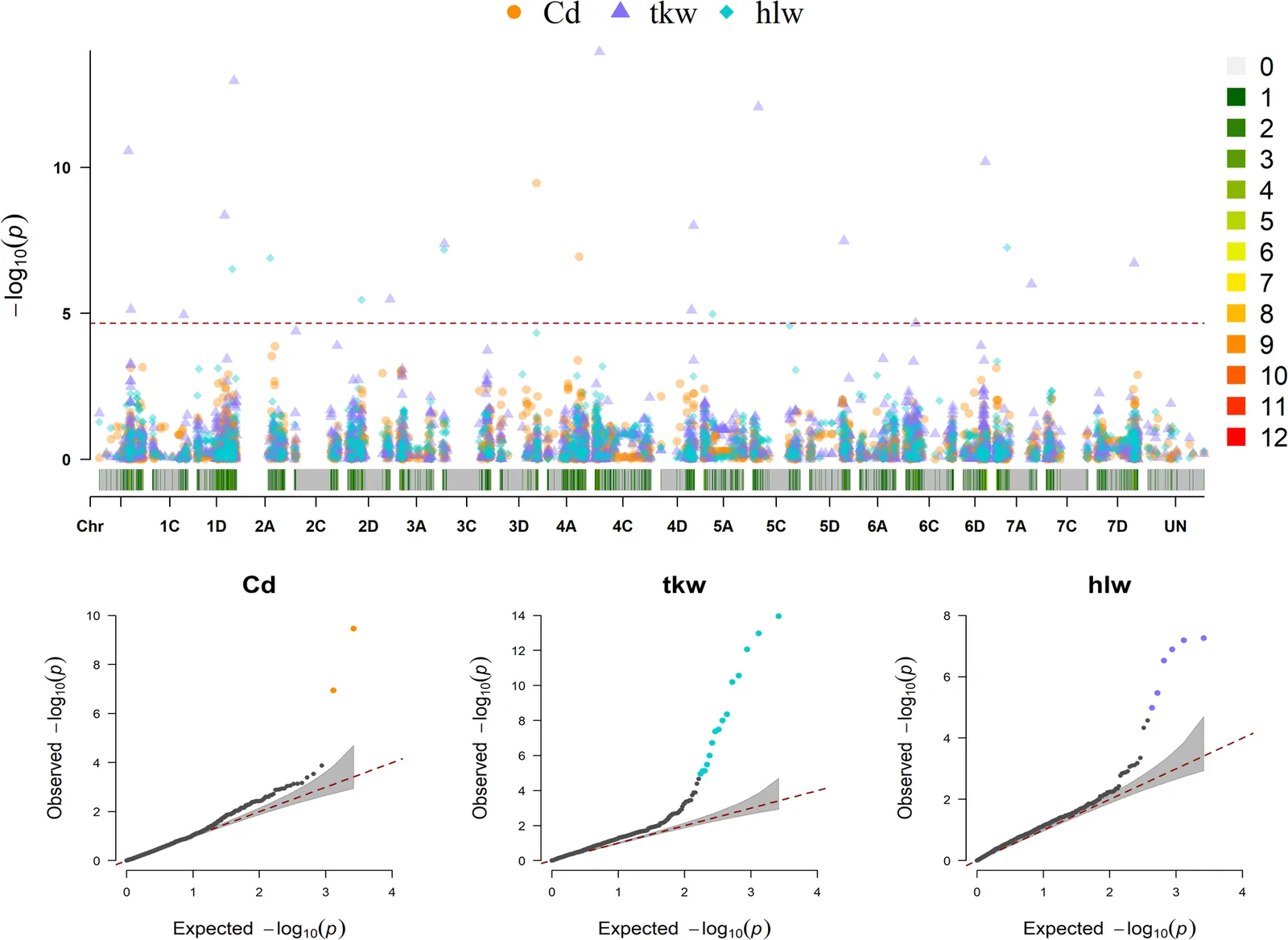
Manhattan plots for genome wide association studies for 596 Nordic spring oat breeding lines analysed in Svalöv, Southern Sweden, for cadmium grain content, hectolitre weight (hlw), and thousand kernel weight (tkw), and corresponding quantile-quantile (QQ) plots of the optimal model (MLMM + Q5 for Cd grain content, FarmCPU + Q5 for hlw, BLINK + Q5 for tkw) with the 95% confidence interval. The red horizontal dashed line represents the Bonferroni correction threshold (α- level 0.05). The x-axis shows the 21 oat chromosomes plus an unassigned chromosome (UN) with physical positions and the SNP density, the y-axis shows the -log10(p-value). Coloured boxes on the right-hand side of the Manhattan plot represents the SNP density

For hlw, six significant marker trait associations (MTAs) were found located on six chromosomes, for tkw 15 MTAs on twelve chromosomes were found and for Cd grain content two MTAs located on two chromosomes were detected (Fig. 4; Table 4).

**Table 4:** Significant marker trait association identified in genome-wide association studies for 596 Nordic spring oat breeding lines analysed in Svalöv, Southern Sweden, for hectolitre weight, thousand kernel weight, and Cadmium grain content

SNP	Chr	Position [bp]	LOD	MAF	Effect	PVE [%]
Hectolitre weight						
GMI_ES01_c1223_200	1D	421,090,061	6.523	0.2324	0.2964	0.000
ZOT000452	2A	293,849,688	6.889	0.2139	0.2676	0.003
GMI_ES_LB_11007	2D	171,308,812	5.469	0.0369	-0.4241	0.000
ZOT001308	3C	17,890,456	7.184	0.2919	0.2817	0.939
LOT007219	5A	106,211,047	4.983	0.0805	-0.3225	0.000
LOT105629	7A	130,654,765	7.258	0.2878	-0.2315	0.000
Thousand kernel weight						
LOT075719	1A	350,035,132	10.561	0.0277	1.3675	3.775
LOT024937	1A	381,507,054	5.130	0.2114	0.4424	0.796
ZOT003330	1C	375,743,208	4.947	0.2424	-0.3329	0.491
LOT086329	1D	327,509,650	8.355	0.2089	0.4816	0.860
GMI_ES17_lrc20172_521	1D	439,461,998	12.967	0.0747	-0.9255	2.362
GMI_GBS_7372	2D	513,096,949	5.479	0.2139	0.3609	0.945
ZOT001308	3C	17,890,456	7.378	0.2919	0.5216	0.394
ZOT002828	4C	54,993,328	13.955	0.3070	0.6149	1.155
LOT125999	4D	374,128,308	5.110	0.4161	0.3761	0.997
GMI_ES02_c12708_687	4D	396,770,086	8.003	0.0663	0.9263	4.370
ZOT000683	5C	67,587,133	12.065	0.0302	1.7271	10.568
LOT057602	5D	411,430,003	7.480	0.2081	-0.4765	0.677
GMI_DS_LB_6200	6D	268,248,165	10.190	0.3448	0.5426	0.963
LOT067097	7A	421,012,546	6.005	0.0570	0.6681	1.337
GMI_ES03_c13331_202	7D	440,937,804	6.719	0.0495	-0.7840	1.260
Cadmium grain content						
GMI_ES17_c3665_257	3D	432,535,695	9.464	0.0277	-0.0114	34.690
GMI_ES14_c17336_70	4A	381,225,958	6.938	0.0201	0.0107	27.920

Chr – chromosome, Position – physical position based on ‘Sang’ reference genome (Kamal et al. 2022); LOD – logarithm of odds, MAF – minor allele frequency, Effect – SNP effect, PVE –phenotypic variation explained by a single SNP marker

#### Hectolitre weight

The six MTAs associated with hlw were distributed across six chromosomes, namely 1D, 2A, 2D, 3C, 5A and 7A (Fig. 4). LOD values ranged from 5.47 to 7.26 (Table 4). The three SNPs GMI_ES01_c1223_200 (1D, 421 Mbp), ZOT000452 (2A, 293 Mbp), and ZOT001308 (3C, 17.890 Mbp) had positive effects on hlw (Table 4, Suppl. Figure 4). The remaining three SNPs (GMI_ES_LB_11007, LOT007219, LOT105629) had negative effects on hlw. All six SNPs explained less than 1% of the phenotypic variation (Table 4). The allele status at the corresponding SNP for each line as well as the corresponding BLUE values can be found in Table S3.

#### Thousand kernel weight

**Fig. 5:**
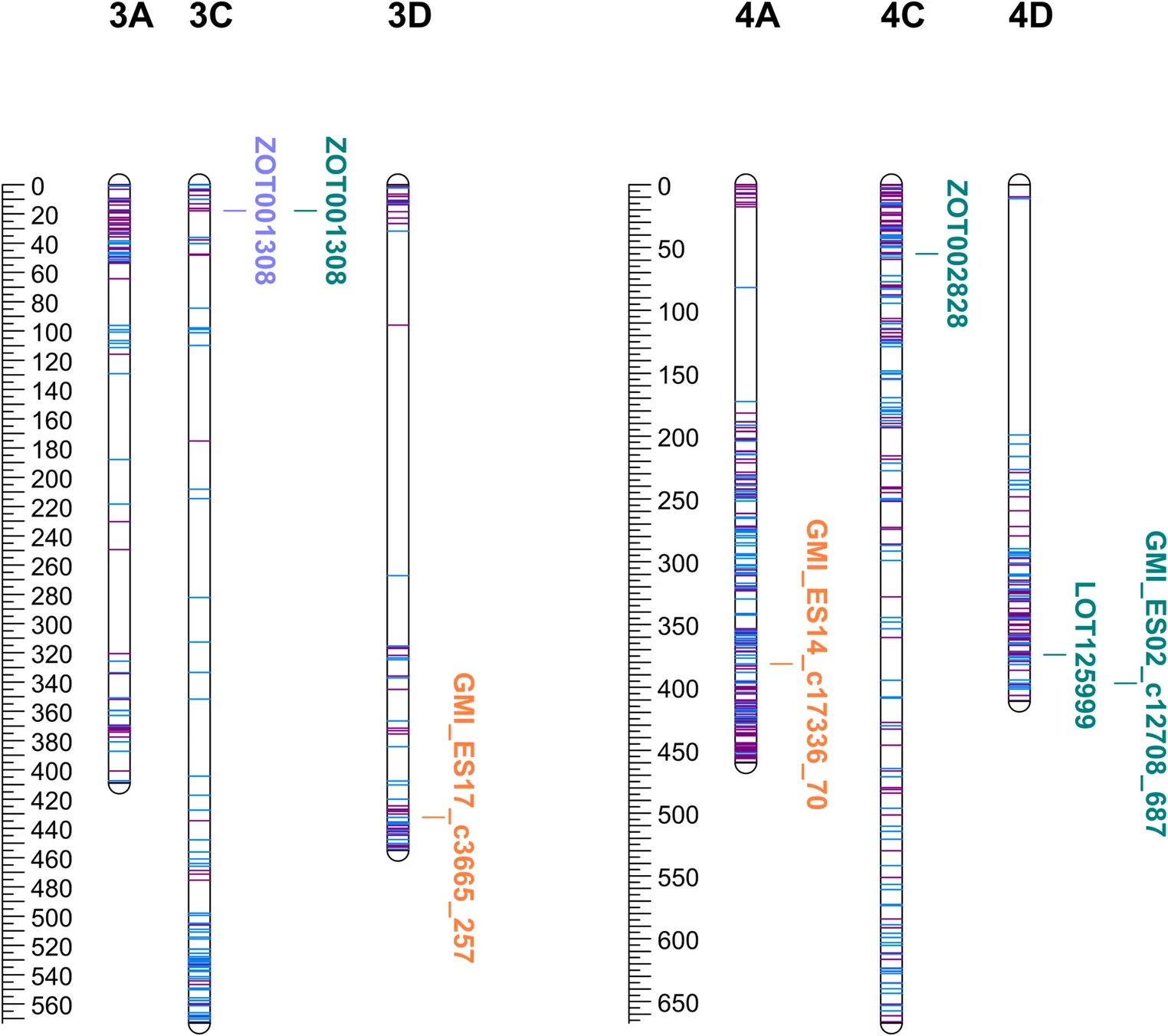
Chromosome maps of oat chromosomes 3A, 3C and 3D and 4A, 4C, and 4D showing significant marker trait associations identified in 596 Nordic spring oat breeding lines for the traits hectolitre weight (lilac), thousand kernel weight (green), and Cadmium grain content (orange)

Fifteen SNPs were significantly associated with tkw located across twelve chromosomes (Fig. 4; Table 4). The first two MTAs were located on chromosome 1A at 350 Mbp (LOT075719) and 381 Mbp (LOT024937). The first SNP (LOT075719) explained 3.78% of the phenotypic variation and had a LOD value of 10.56. On chromosome 1C, SNP ZOT003330 located at 375 Mbp had a negative effect on tkw, but only explained 0.49% of the phenotypic variation. Two MTAs were located on chromosome 1D, at 32 Mbp (LOT086329) and 439 Mbp (GMI_ES17_lrc20172_521). The latter SNP had a LOD value of 12.97 and explained 2.36% of the phenotypic variation. The effect on tkw, however, was negative (Table 4, Suppl. Figure 5). The two SNPs located on chromosomes 2D (GMI_GBS_7372, 513 Mbp) and 3C (ZOT001308, 17Mbp) explained only 0.95 and 0.39% of the phenotypic variation, respectively. However, SNP ZOT001308 is interesting nonetheless, since this SNP was also significant for hlw (Fig. 5). On chromosome 4C, the MTA with the highest LOD value (13.96) was located at 54 Mbp and explained 1.16% of the phenotypic variation with a positive effect on tkw (Table 4). Two MTAs were located on chromosome 4D at 374 Mbp (LOT125999) and 396 Mbp (GMI_ES02_c12708_687) with LOD values of 5.11 and 8.0, respectively, with positive effects on tkw (Table 4). SNP marker ZOT000683 is located on chromosome 5C at 67 Mbp, was highly significant (LOD 12.07) and explained 10.57% of the phenotypic variation with a positive effect on tkw (1.73 g; Table 4). On chromosomes 5D and 6D there was one MTA, respectively, located at 411 Mbp and 268 Mbp. The last two MTAs were located on chromosomes 7A at 421 Mbp (LOT067097) and 7D at 440 Mbp (GMI_ES03_c13331_202) with LOD values ranging between 6.0 and 6.7 and explained phenotypic variations of 1.26 and 1.34% (Table 4). The allele status at the corresponding SNP for each line as well as the corresponding BLUE values can be found in Table S4.

#### Cadmium grain content

**Fig. 6:**
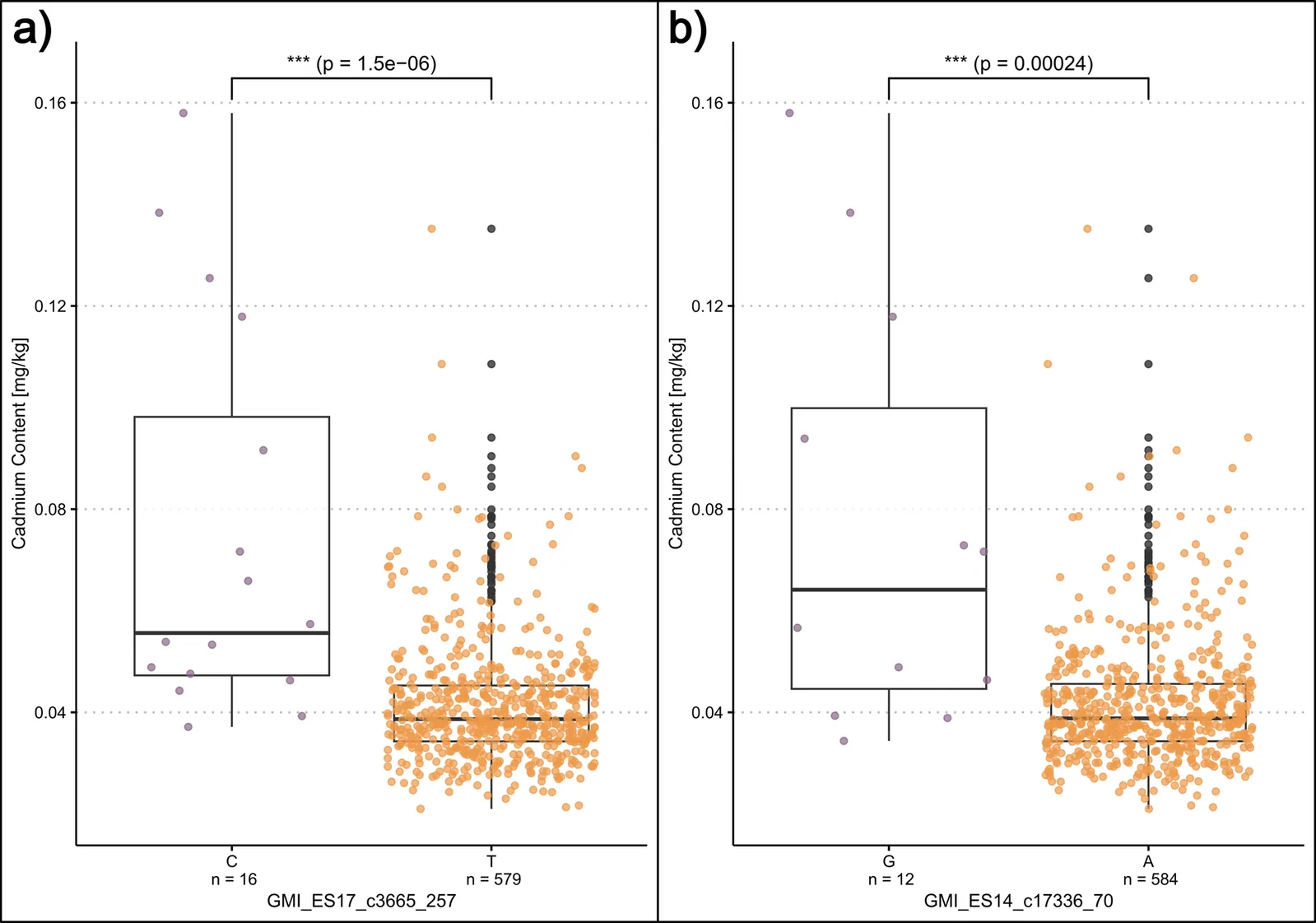
Allele effect on Cadmium grain content for MTAs located on chromosome (**a**) 3D at 432,535,695 bp (GMI_ES17_c3665_257) and (**b**) 4A at 381,225,958 bp (GMI_ES14_c17336_70). Lilac and orange dots represent values of Cd grain content for each individual line. Grey dots represent outliers. Asterisks represent significance thresholds of the Wilcox test between groups, n indicates the number of observations per group

For Cd grain content two significant MTAs were identified (Fig. 5). The first MTA was located on chromosome 3D at 432,535,695 bp (GMI_ES17_c3665_257) and had a LOD value of 9.46. It had a significant effect on Cd grain content (-0.0114 mg/ kg) and explained 34.69% of the phenotypic variation. (Table 4; Fig. 6a, Table S5.1).

The second MTA was located on chromosome 4A at 381,225,958 bp (GMI_ES14_c17336_70). It had a significant effect on Cd grain content, as well, (0.0107 mg/ kg) and explained 27.92% of the phenotypic variation (Table 4; Fig. 6b, Table S5.1).

The factorial ANOVA revealed a significant interaction of the two SNPs, which explained 2.2% of the total variation (Table S5.1). SNP marker GMI_ES17_c3665_257 explained 2.4% and SNP marker GMI_ES14_c17336_70 explained 4.8°% of the total variation (Table S5.1). The analysis of the simple effects showed that SNP marker GMI_ES17_c3665_257 had a greater effect on Cd grain accumulation than SNP marker GMI_ES14_c17336_70 (Table S5.2 and S5.3). The proportion of total variance explained for a specific factor is calculated by dividing the factor’s sum of squares by the total sum of squares. However, the differences in the proportion of total explained variation between the ANOVA results and the GWAS results, stems from the fact that GAPIT incorporates the magnitude of the marker effect as well as the MAF of a marker into the calculation (Wang and Zhang 2021)

Of the 596 genotypes, 573 carry both favourable alleles (Table S6). Lines carrying both favourable alleles showed the highest average reduction in Cd grain content compared to lines carrying no favourable alleles. Six and eleven lines carried only one of the favourable alleles, respectively. These lines showed significantly reduced average Cd grain content values compared to lines carrying no favourable alleles (Table S5 and S6). The favourable alleles are fixed in Lantmännen’s breeding germplasm pool and hence, many lines have made it to the market as registered cultivars and are publicly available (Table S6)

In the proximity of the significant SNP on chromosome 3D (432.535 Mbp; GMI_ES17_c3665_257) several putative candidate genes were identified. The first gene, AVESA.00010b.r2.3DG0562400 is located at 413 Mbp and codes for a heavy-metal associated protein. The primer pair designed for this candidate gene amplified a product with a size of 4503 bp (Table S1, Suppl. Figure 7a). The sequences showed polymorphisms at bp positions 1716 to 1731 (Suppl. Figure 8a). Local LD between the significant SNP and the region of the first candidate gene was strong with D’ = 1; however, the r² value for the proposed gene located at 413 Mbp was almost 0 (Supp. Figure 6a).

The significant SNP was also in strong LD with two SNPs upstream (LOT122068 at 428 Mbp and LOT077610 at 430 Mbp), with D’ = 1 and r² ranging from 0.36 to 0.58 (Supp. Figure 6a). In this 4 Mbp region, there are 37 high confidence genes annotated on the Sang genome. Several code for protein kinases, two for Pentatricopeptide repeat (PPR) superfamily proteins, one for a plant AT-rich sequence and zing-binding (PLATZ) transcription factor and one for a wall-associated kinase (WAK) family protein. Since the latter was suggested by Tanhuanpää et al. (2007) as a candidate gene for Cd accumulation in oats, this gene (AVESA.00010b.r2.3DG0565980 at 430 Mbp) was sequenced as a second possible candidate gene. The primer pair designed for candidate gene AVESA.00010b.r2.3DG0565980 (430 Mbp) amplified a product with a size of 4976 bp (Table S1, Suppl. Figure 7b). No amplicon was observed for line SOA026. Therefore, another reverse primer (3D_430-r1) was tested, which was supposed to yield an amplicon size of 4514 bp (Table S1). However, the observed amplicon had a size of about 1245 bp (Suppl. Figure 7c). Sequencing and BLAST search results against the Sang genome did not map the amplicon to any specific region, hence, this sequence was excluded from further analysis. The other seven genotypes showed no polymorphisms in the sequenced region, except for genotype Active at bp position 3720 and 3721 bp (Suppl. Figure 8b).

The significant SNP GMI_ES14_c17336_70 on chromosome 4A at 381.225 Mbp is located just next to the gene AVESA.00010b.r2.4AG0634750 (381.222 Mbp), which codes for a heavy metal transport/ detoxification superfamily protein. The LD analysis showed that the significant SNP is in strong LD (D’ = 1) with several SNPs surrounding it, one SNP is even located within the region of the candidate gene. However, the corresponding r²-values were very low (Suppl. Figure 6b). The primer pair designed to target the gene on chromosome 4A amplified a product with a size of 4339 bp (Table S1). However, no product was amplified in five out of the eight lines (Suppl. Figure 7d).

## Discussion

Cd is a heavy-metal that accumulates in the soil, is taken up by the plant through the root system and subsequently distributed into above ground plant parts (Abedi and Mojiri 2020; Hu et al. 2022). The accumulated amount in the plant was shown to be influenced for example by the amount of Cd in the soil, soil properties, environmental conditions, and the crop genotype (Eriksson 1990; Eriksson et al. 1996; Eurola et al. 2003; Arao and Ishikawa 2006; Liu et al. 2015). High concentrations of Cd in the plant lead to oxidative stress, antioxidant disruption, nutrient imbalance and impairment of the photosynthetic activity, ultimately negatively impacting yield components and yield (Anjum et al. 2015; Azhar et al. 2019; Mei et al. 2022). Additionally, Cd negatively affects human health (Chunhabundit 2016). With the increased demand of plant-based products, it is crucial to reduce Cd uptake through food products by producing cereal grains with low Cd concentrations in the grain.

The current study investigated 620 Nordic spring oat breeding lines for their Cd grain content tested in field trials in Sweden between 2015 and 2021 to identify genomic regions associated with Cd grain accumulation. Additionally, since hectolitre weight (hlw; test weight) and thousand kernel weight (tkw) are important measures for oat quality, we also looked at these two traits (Forsberg and Reeves 2015).

There was a significant negative correlation between Cd grain content and tkw, suggesting that Cd concentration in the grain reduced kernel size and potentially yield. Confirming previous reports of Cd impairing yield and yield components (Anjum et al. 2015; Azhar et al. 2019; Mei et al. 2022). However, the correlation was low. There was no correlation between Cd grain content and hlw. Hence, these results suggest it is possible to breed for oat cultivars with low Cd grain concentration, high tkw, and high hlw.

After filtering for minor allele frequency, the GWAS was conducted for 596 genotypes on 2613 SNPs. This number sounds certainly low especially compared to the big genome size of *Avena sativa*. The investigated oat panel was genotyped with the 7k Illumina oat SNP genotyping array as a routine method within the Lantmännen breeding programme. Many of the SNPs could not be mapped against the ‘Sang’ reference genome, which might be due to the many structural variations within the *Avena* genome. Additionally, as the panel is a breeding population many SNPs were also excluded because they were not polymorphic and therefore not informative. With more SNPs, more QTL and especially minor QTL could have been identified. However, the high mean effects of the MTAs associated with Cd grain accumulation support the validity of the identified MTAs within this study, at least for SNPs associated with Cd accumulation. SNPs associated with tkw and hlw did not show as high effects, nonetheless identified QTL corresponded to previously reported regions. The putatively new QTL might be treated with caution and need further validation. Even though the low SNP number can be seen as a drawback, the high phenotypic depth is the strength of this study.

GWAS for hlw and tkw revealed six and 15 significant MTAs, respectively. Two MTAs were located on chromosome 1A at 350 to 381 Mbp associated with tkw. Yan et al. (2023) reported SNPs on chromosome 1A associated with tkw and grain weight. Their SNPs were located at 17.56 Mbp and 65.89 Mbp and at 347.22 Mbp based on the ‘Sanfensan’ reference genome. Suggesting our MTAs and the MTA reported by Yan et al. (2023) located on the long arm of chromosome 1A are the same. One and three MTAs were located on chromosomes 1C (375.74 Mbp) and 1D (327.5 to 439.46 Mbp). No QTL located on chromosome 1C were reported in previous studies. Esvelt Klos et al. (2021) reported a QTL on chromosome 1D at 118.9 cM based on the consensus map of Chaffin et al. (2016) associated with hlw. This locus could be the same as the one reported in the present study, however, to verify it, further studies are necessary.

On chromosomes 2A (293.54 Mbp) and 2D (171.31 Mbp and 513.1 Mbp), we detected one and two MTAs associated with hlw and tkw. QTL for hlw and tkw on chromosomes 2A and 2D were also identified by Esvelt Klos et al. (2021) at 34 cM and 142 cM, respectively. Tinker et al. (2022) found a QTL on chromosome 2D at 150 to 173 Mbp based on the ‘Sang’ reference genome. Our MTA at 171.31 Mbp is located right in their interval and therefore probably represents the same QTL.

The MTAs identified on chromosome 3C are particularly interesting, since the same SNP marker was significantly associated with hlw and tkw (ZOT001308, 17,890,456 Mbp). Since no QTL has been reported on this chromosome yet, this could be a putatively new QTL.

Yan et al. (2023) reported two QTL on chromosome 4C located at 159.47 Mbp and 674.57 Mbp based on the ‘Sanfensan’ reference genome. Our MTA was located at 54.99 Mbp and therefore most likely represents an independent locus. Several studies have identified QTL associated with grain weight, grain shape or hlw located on chromosome 4D (De Koeyer et al. 2004; Esvelt Klos et al. 2021; Zimmer et al. 2021; Tinker et al. 2022; Ardayfio et al. 2025). De Koeyer et al. (2004) reported a QTL associated with kernel weight and hlw near the N1 locus on linkage group TM_5. The N1 locus, coding for hulless kernels in oat, was mapped to chromosome 4D flanked by SNP markers GMI_ES14_c19259_657, GMI_GBS_86198, GMI_ES22_c7478_431 and GMI_ES17_c5923_221 (Ubert et al. 2017). Markers GMI_GBS_86198 and GMI_ES17_c5923_221 are located at 405.68 and 410.39 Mbp, respectively, on the ‘Sang’ genome. The other two markers could unfortunately not be mapped or were not present in our panel. In a GWAS, Peng et al. (2022) identified the N1 locus on chromosome 4D at 450.96 Mbp on the ‘Sanfensan’ reference genome. Esvelt Klos et al. (2021) detected a QTL associated with hlw and tkw at 195.7 to 212.1 cM, stating it corresponds to the QTL reported by De Koeyer et al. (2004) near the N1 locus. On the ‘Sang’ reference genome, Tinker et al. (2022) reported the N1 locus to be located around 411 Mbp. Next to other yield parameters, they reported significant associations for tkw in an interval between 407 and 415 Mbp. In the present study, two MTAs were significantly associated with tkw located on chromosome 4D at 374.13 and 396.77 Mbp. If our QTL is associated with the N1 locus as well, cannot be stated conclusively, however, it certainly is in close proximity to it.

The present study identified one MTA each on chromosomes 5A (106 Mbp), 5C (67 Mbp), 5D (411 Mbp), and 6D (268 Mbp), respectively. No QTL associated tkw or hlw have been reported on chromosome 5A or 6D so far. QTL on chromosome 5C associated with tkw were reported by Oliveira et al. (2025) located at 364.66 Mbp on the ‘OT3098’ oat reference genome (v2) in a population adapted to sub-tropic conditions. However, their reported MTA and ours most likely represent two independent regions. On chromosome 5D, two QTL associated with tkw and hlw have been reported located at 42.13 Mbp (‘Sanfesan’ reference genome, Yan et al. 2023) and at 26.4 cM (Esvelt Klos et al. 2021), respectively. Again, our MTA most likely represents a putatively new QTL.

The two MTAs detected on chromosomes 7A (130 Mbp and 421 Mbp) represent two independent loci. Ardayfio et al. (2025) reported a QTL for hlw located at 253.79 Mbp on the ‘Sang’ reference genome. Additionally, Tinker et al. (2022) reported a QTL for tkw at 60 to 72 Mbp (‘sang’ reference genome), which overlapped with the location of a gene copy of *Vrn3*. However, our MTAs and the loci reported by Ardayfio et al. (2025) and Tinker et al. (2022) probably represent independent loci. Lastly, on chromosome 7D, QTL for hlw were reported at 305.06 Mbp (‘Sanfensan’ reference genome; Yan et al. 2023) and at 85.2 to 88.7 cM (Esvelt Klos et al. 2021). Our QTL on chromosome 7D was located at 440 Mbp and might be independent from the previously reported ones. Interestingly, a gene copy of *Vrn3* is located in close proximity at 469 Mbp and might be associated with the QTL identified in the present study (Tinker et al. 2022).

The main goal of this study, was to identify QTL associated with Cd grain accumulation in oat. Within the panel of 596 Nordic spring oat breeding lines, we identified two major QTL associated with Cd accumulation in the grain located on chromosomes 3D at 432.54 Mbp and 4A at 381.23 Mbp.

So far, only one other study was done on this subject. Tanhuanpää et al. (2007) identified a major QTL for Cd grain accumulation in oats in a cross of ‘Aslak’ x ‘Salo’, with the latter parent tending to accumulate higher amounts of Cd. Their QTL was significantly associated with RAPD markers OPAF20_1350_ and OPAF15_960_. At the time of their study, they were not able to identify the exact location of their QTL, however, they proposed a wall associated kinase (WAK3) to be a likely candidate gene.

A blast search against the ‘Sang’ reference genome for the two markers OPAF20_1350_ and OPAF15_960_ mapped these two markers on chromosomes 7A and 7D. Marker OPAF20_1350_ showed a 99% query coverage with a region on chromosome 7A at 57.96 Mbp and a 91% query coverage with a region on chromosome 7D at 479.08 Mbp. The region on chromosome 7A partly covers a gene coding for a wall-associated receptor kinase protein, the one on 7D for a WAK, confirming the proposed candidate gene of Tanhuanpää et al. (2007).

Marker OPAF15_960_ showed a 97% query coverage on chromosome 7A at 65.71 Mbp and a query coverage of 99% on chromosome 7D at 472.45 Mbp. These two regions overlapped with genes coding for B3 domain containing proteins. If the previously reported QTL is located on chromosome 7A or 7D, or if these regions represent gene copies, cannot be conclusively said. However, none of the two QTL in the present study corresponds to the QTL reported by Tanhuanpää et al. (2007) and therefore, represent two putatively new major QTL associated with Cd grain accumulation in oats.

About 19 Mbp upstream of our MTA on chromosome 3D (432 Mbp), a gene is located coding for a heavy metal-associated protein (HMP) (AVESA.00010b.r2.3DG0562400, 413 Mbp; ‘Sang’ reference genome). Our second MTA on chromosome 4A (381 225 958 bp) is located less than 2000 bp downstream of a gene belonging to a heavy metal transport/ detoxification superfamily (AVESA.00010b.r2.4AG0634750, 381,222,231–381,224,041 bp). A blast search of the gene sequences against the ‘OT3098’ oat reference genome (v2), revealed these two genes code for heavy metal-associated isoprenylated plant proteins (HIPPs), HIPP6 (3D, AVESA.00001b.r3.3Dg0002386) and HIPP7 (4A, AVESA.00001b.r3.4Ag0002908), respectively. HIPPs have been shown to be exclusive to plants and to be involved in responses to abiotic and biotic stress, but most importantly to heavy metal homeostasis and detoxification (De Abreu-Neto et al. 2013). They are metal chaperons, which consist of a heavy-metal associated (HMA) binding domain and a C-terminal isoprenylation motif (De Abreu‐Neto et al. 2013). Their involvement in Cd detoxification or tolerance was shown in several plant species like *Arabidopsis thaliana* (Suzuki et al. 2002; De Abreu‐Neto et al. 2013; Li et al. 2020), *Haynaldia villosa* (Zhang et al. 2020a), *Camellia sinensis* (Wei et al. 2023), and *Oryza sativa* (De Abreu‐Neto et al. 2013; Li et al. 2020; Cao et al. 2022). Based on their annotations and proximity to the respective significant SNP, these two genes were considered possible candidate genes. In order to verify this assumption, five lines carrying the favourable alleles and showing low values for Cd grain content, and three lines carrying the unfavourable alleles with high values of Cd grain content were chosen for sequencing the regions of these genes. For both genes there were no polymorphisms in the sequence of the gene, not within the eight sequenced lines, nor when compared to the ’Sang’ sequence. The sequences were blasted against other oat reference genomes at GrainGenes, as well as at the NCBI database (data not shown). The gene sequences seem to be highly conserved within the *Avena sativa* genome, but also across different Poaceae genera like *Triticum*, *Brachypodium* and *Panicum*.

For gene AVESA.00010b.r2.4AG0634750, the PCR showed amplified products in only three lines, i.e. SOA369, SOA372 and Active. We compared the reference sequence of ‘Sang’, which we used to design the primers, with five other oat reference genomes, i.e. Aslak, Delfin, Bingo, Legget and Williams. The Blast search showed that the latter three lacked the second half of the region, where the reverse primer was supposed to bind. We hypothesized that this might also be the case in our lines. Hence, the primer could not bind and no product was amplified. However, based on the current data, we cannot either confirm nor completely dismiss this hypothesis. Nevertheless, these results indicate that there is a widespread structural polymorphism at this region, underlining the well-known structural variants in the *Avena* genome.

Looking at localised LD for chromosome 4A, we see that the significant SNP and the flanking SNP upstream, which is located within the gene, are in strong LD, i.e. high D’. However, the r² value was basically zero. This can be explained with the low MAF of the SNPs in question. The significant SNP had a MAF = 2.01%, and did therefore just make the MAF cut-off of 2% for being included in the GWAS. The flanking SNP, on the other hand, had a MAF below 2%. Now, r² gives information about the correlation of two genetic loci, or their variants. If r² is close to 1, one variant perfectly predicts the other variant and vice versa. In our case, the favourable allele of the significant SNP is always found with the major allele of the SNP in the gene, but not vice versa, which makes for the low r² value. Nonetheless, LD seems to be strong around the significant SNP and the proposed candidate gene. Since the three lines that had an amplified product, were all lines that carried the positive allele and showed low Cd grain contents, it was not possible at this point to confirm nor dismiss this gene as a candidate gene. However, the significant SNP is in very close proximity (about 2000 bp) and within the sequenced region and therefore still presents a strong candidate. If there are polymorphisms outside of the sequenced region that influence transcription of this gene or if another gene entirely is associated with the phenotype has to be investigated further.

There were polymorphisms in the amplified sequence of the promoter region for gene AVESA.00010b.r2.3DG0562400 (3D, 413 Mbp). However, these polymorphisms did not clearly translate into differences separating the lines with low Cd grain contents from the lines with high Cd grain contents. Similar to the candidate gene on chromosome 4A, the significant SNP and the region of the candidate gene showed moderate to high D’ values, indicating this region is inherited together. Again, MAFs were low, leading to low to moderate r² values. However, based on the inconclusive sequencing results, we presume this is not the underlying gene.

The second candidate gene (AVESA.00010b.r2.3DG0565980) for the significant MTA on chromosome 3D codes for a wall-associated kinase protein (WAK). On the ‘OT3098’ oat reference genome (v2) this gene (AVESA.00001b.r3.3Dg0002529) is annotated as WAK3 and located at 454 Mbp. WAKs usually consist of three domains; an extracellular domain with an epidermal growth factor (EGF), which is less conserved, a transmembrane domain, and an intracellular serine/threonine protein kinase domain, which is highly conserved (He et al. 1996, 1999; Verica and He 2002). They are involved in signal transduction between the cell wall and the cytoplasm and have been described to be vital for plant development (Wagner and Kohorn 2001; Kohorn et al. 2006). Additionally, WAKs have been reported to be involved in responses to biotic and abiotic stress, as well as heavy-metal handling (He et al. 1998; Sivaguru et al. 2003; Hou et al. 2005; Yin and Hou 2017; Xia et al. 2018). Lastly, Tanhuanpää et al. (2007) as well suggested a WAK3 as their candidate gene. Five low Cd and two high Cd lines were sequenced for this gene region, but the sequences showed no polymorphisms that would support this gene as a candidate. Hence, we dismiss this as the underlying gene.

Other candidate genes that are in close proximity to the significant SNP are genes coding for zinc transporters (428 Mbp), PLATZ transcription factors (431 Mbp) and PPR superfamily proteins (432 Mbp). Zinc transporters have been reported to be involved in handling heavy-metal stress (Tao and Lu 2022). A study in *Populus trichocarpa* showed that PLATZ genes are upregulated in response to heavy-metal and in particular to Cd stress (Ma et al. 2023). Similarly, an upregulated or differential expression upon Cd stress of PPR proteins was shown in *Oryza sativa*, *Sorghum bicolor*, and *Arabidopsis thaliana* (Ahsan et al. 2007; Roy et al. 2016; Sferra et al. 2023). These three genes represent strong candidate genes that have to be investigated further. This could be either done by developing further markers to sequence each amplicon, or by sequencing the whole region surrounding the significant SNPs on chromosome 3D and 4A.

## Conclusion

This study represents the first GWAS for Cd grain accumulation on oats. Within a panel of 596 Nordic spring oat breeding lines, we identified two major QTL associated with cadmium grain accumulation on chromosomes 3D (432.54 Mbp) and 4A (381.23 Mbp). These loci represent putatively novel genomic regions from previously reported QTL. No candidate genes could be conclusively confirmed at this point.

Additionally, we identified MTAs for thousand kernel weight and hectolitre weight, several of which validate previously reported QTL, while others represent putatively new loci. The weak correlation between Cd content and grain quality traits suggests breeding of oat cultivars with low Cd grain content is possible without compromising grain quality traits.

## Supplementary Information

Below is the link to the electronic supplementary material.

Supplementary Material 1

Supplementary Material 2

Supplementary Material 3

Supplementary Material 4

Supplementary Material 5

Supplementary Material 6

Supplementary Material 7

Supplementary Material 8

Supplementary Material 9

## Data Availability

The datasets on phenotypic traits are available under http://dx.doi.org/10.5447/ipk/2026/1. The genotypic data on the spring oat lines is available under https://doi.org/10.5281/zenodo.20397021. Sequencing data was deposited at NCBI; accession numbers can be found in Supplementary Table S2. All data supporting the findings of this study are available within the paper and its Supplementary Information.

## References

[CR1] Abedi T, Mojiri A (2020) Cadmium Uptake by Wheat (*Triticum aestivum* L.): An Overview. Plants 9:500. 10.3390/plants9040500 PMC7238532 32295127

[CR2] AbuHammad WA, Mamidi S, Kumar A et al (2016) Identification and validation of a major cadmium accumulation locus and closely associated SNP markers in North Dakota durum wheat cultivars. Mol Breed 36:112. 10.1007/s11032-016-0536-1

[CR3] Ahsan N, Lee S-H, Lee D-G et al (2007) Physiological and protein profiles alternation of germinating rice seedlings exposed to acute cadmium toxicity. C R Biol 330:735–746. 10.1016/j.crvi.2007.08.001 17905393

[CR4] Anjum SA, Tanveer M, Hussain S et al (2015) Cadmium toxicity in Maize (*Zea mays* L.): consequences on antioxidative systems, reactive oxygen species and cadmium accumulation. Environ Sci Pollut Res 22:17022–17030. 10.1007/s11356-015-4882-z 26122572

[CR6] Arao T, Ishikawa S (2006) Genotypic Differences in Cadmium Concentration and Distribution of Soybean and Rice. Jpn Agric Res Q JARQ 40:21–30. 10.6090/jarq.40.21

[CR5] Arao T, Ae N, Sugiyama M, Takahashi M (2003) Genotypic differences in cadmium uptake and distribution in soybeans. Plant Soil 251:247–253. 10.1023/A:1023079819086

[CR7] Ardayfio NK, Kinney C, Li D et al (2025) Genome-wide association studies reveal genetic control of nutritional quality, milling traits, and agronomic characteristics in oat (*Avena sativa* L). Plant Genome 18:e70060. 10.1002/tpg2.70060 PMC12234416 40623918

[CR8] Azhar M, Zia-ur-Rehman M, Murtaza G, Ahmad E (2019) Effect of increasing levels of applied cadmium on growth, biochemical attributes and micronutrient uptake by wheat and rice. Pak J Agric Sci 56:205–214. 10.21162/PAKJAS/19.6755

[CR9] Ballabio C, Jones A, Panagos P (2024) Cadmium in topsoils of the European Union – An analysis based on LUCAS topsoil database. Sci Total Environ 912:168710. 10.1016/j.scitotenv.2023.168710 38008327

[CR10] Bates D, Mächler M, Bolker B, Walker S (2015) Fitting Linear Mixed-Effects Models Using lme4. J Stat Softw 67. 10.18637/jss.v067.i01

[CR12] Benitez ER, Hajika M, Yamada T et al (2010) A Major QTL Controlling Seed Cadmium Accumulation in Soybean. Crop Sci 50:1728–1734. 10.2135/cropsci2009.11.0664

[CR11] Benitez ER, Hajika M, Takahashi R (2012) Single-Base Substitution in P1B-ATPase Gene Is Associated with a Major QTL for Seed Cadmium Concentration in Soybean. J Hered 103:278–286. 10.1093/jhered/esr123 22268161

[CR13] Browning BL, Zhou Y, Browning SR (2018) A One-Penny Imputed Genome from Next-Generation Reference Panels. Am J Hum Genet 103:338–348. 10.1016/j.ajhg.2018.07.015 PMC6128308 30100085

[CR14] Cao HW, Zhao YN, Liu XS et al (2022) A metal chaperone *OsHIPP16* detoxifies cadmium by repressing its accumulation in rice crops. Environ Pollut 311:120058. 10.1016/j.envpol.2022.120058 36041567

[CR15] Çatav ŞS, Genç TO, Oktay MK, Küçükakyüz K (2020) Cadmium Toxicity in Wheat: Impacts on Element Contents, Antioxidant Enzyme Activities, Oxidative Stress, and Genotoxicity. Bull Environ Contam Toxicol 104:71–77. 10.1007/s00128-019-02745-4 31748863

[CR16] Chaffin AS, Huang Y, Smith S et al (2016) A Consensus Map in Cultivated Hexaploid Oat Reveals Conserved Grass Synteny with Substantial Subgenome Rearrangement. Plant Genome 9. 10.3835/plantgenome2015.10.0102. :plantgenome2015.10.0102 27898818

[CR17] Chen L, Wan H, Qian J et al (2018) Genome-Wide Association Study of Cadmium Accumulation at the Seedling Stage in Rapeseed (Brassica napus L). Front Plant Sci 9:375. 10.3389/fpls.2018.00375 PMC5917214 29725340

[CR18] Chunhabundit R (2016) Cadmium Exposure and Potential Health Risk from Foods in Contaminated Area, Thailand. Toxicol Res 32:65–72. 10.5487/TR.2016.32.1.065 PMC4780235 26977260

[CR19] Cieśliński G, Van Rees KCJ, Huang PM et al (1996) Cadmium uptake and bioaccumulation in selected cultivars of durum wheat and flax as affected by soil type. Plant Soil 182:115–124. 10.1007/BF00011000

[CR20] Commission Regulation (EU) (2021) 2021/1323 of 10 August 2021 amending Regulation (EC) 1881/2006 as regards maximum levels of cadmium in certain foodstuffs

[CR21] Covarrubias-Pazaran G (2016) Genome-Assisted Prediction of Quantitative Traits Using the R Package sommer. PLoS ONE 11:e0156744. 10.1371/journal.pone.0156744 PMC4894563 27271781

[CR22] Curie C, Cassin G, Couch D et al (2009) Metal movement within the plant: contribution of nicotianamine and yellow stripe 1-like transporters. Ann Bot 103:1–11. 10.1093/aob/mcn207 PMC2707284 18977764

[CR23] De Abreu-Neto JB, Turchetto‐Zolet AC, De Oliveira LFV et al (2013) Heavy metal‐associated isoprenylated plant protein (HIPP): characterization of a family of proteins exclusive to plants. FEBS J 280:1604–1616. 10.1111/febs.12159 23368984

[CR24] De Koeyer DL, Tinker NA, Wight CP et al (2004) A molecular linkage map with associated QTLs from a hulless × covered spring oat population. Theor Appl Genet 108:1285–1298. 10.1007/s00122-003-1556-x 14767596

[CR25] Derakhshani B, Jafary H, Maleki Zanjani B et al (2020) Combined QTL mapping and RNA-Seq profiling reveals candidate genes associated with cadmium tolerance in barley. PLoS ONE 15:e0230820. 10.1371/journal.pone.0230820 PMC7182363 32298285

[CR26] Earl DA, von Holdt BM (2012) STRUCTURE HARVESTER: a website and program for visualizing STRUCTURE output and implementing the Evanno method. Conserv Genet Resour 4:359–361. 10.1007/s12686-011-9548-7

[CR27] EFSA Panel on Dietetic Products, Nutrition and Allergies (NDA) (2010) Scientific Opinion on the substantiation of a health claim related to oat beta glucan and lowering blood cholesterol and reduced risk of (coronary) heart disease pursuant to Article 14 of Regulation (EC) 1924/2006. 10.2903/j.efsa.2010.1885. EFSA J 8:PMC13085209 42004118

[CR30] Eriksson JE (1990) A field study on factors influencing Cd levels in soils and in grain of oats and winter wheat. Water Air Soil Pollut 53. 10.1007/BF00154992

[CR28] Eriksson J, Andersson A, Wenblad A (1990) Cd, Ni and Zn contents of oat grain as related to soil factors and recipitation. Swed J Agric Res 20:81–87

[CR29] Eriksson J, Oborn I, Gunilla J, Arne A (1996) Factors Influencing Cd-Content in Crops. Results from Swedish field investigations. Swed J Agric Res 26:125–133

[CR31] Esvelt Klos K, Yimer BA, Howarth CJ et al (2021) The Genetic Architecture of Milling Quality in Spring Oat Lines of the Collaborative Oat Research Enterprise. Foods 10:2479. 10.3390/foods10102479 PMC8535619 34681528

[CR32] Eurola M, Hietaniemi V, Kontturi M et al (2003) Cadmium Contents of Oats (*Avena sativa* L.) in Official Variety, Organic Cultivation, and Nitrogen Fertilization Trials during 1997 – 1999. J Agric Food Chem 51:2608–2614. 10.1021/jf020893+ 12696945

[CR33] Eurostat (2025) Productions végétales au taux d’humidité standard UE par région NUTS 2

[CR34] Evanno G, Regnaut S, Goudet J (2005) Detecting the number of clusters of individuals using the software structure: a simulation study. Mol Ecol 14:2611–2620. 10.1111/j.1365-294X.2005.02553.x 15969739

[CR35] FAO (2023) Crops and Livestock Products

[CR36] Florijn PJ, Van Beusichem ML (1993) Uptake and distribution of cadmium in maize inbred lines. Plant Soil 150:25–32. 10.1007/BF00779172

[CR37] Forsberg RA, Reeves DL (2015) Breeding Oat Cultivars for Improved Grain Quality. In: Marshall HG, Sorrells ME (eds) Agronomy Monographs. American Society of Agronomy, Crop Science Society of America, Madison, WI, USA, pp 751–775

[CR38] Genchi G, Sinicropi MS, Lauria G et al (2020) The Effects of Cadmium Toxicity. Int J Environ Res Public Health 17:3782. 10.3390/ijerph17113782 PMC7312803 32466586

[CR39] Gezan SA, de Oliveira AA, Galli G, Murray D (2022) ASRgenomics: An R package with Complementary Genomic Functions, Version 1.1.0, VSN International, Hemel Hempstead, United Kingdom, pp 1 - 44.

[CR40] Hall TA (1999) BioEdit: a user-friendly biological sequence alignment editor and analysis program for Windows 95/98/NT. Nucleic Acids Symp Ser 41:95–98

[CR41] He QB, Singh BR (1993) Plant Availability of Cadmium in Soils: I. Extractable Cadmium in Newly and Long-Term Cultivated Soils. Acta Agric Scand Sect B - Soil Plant Sci 43:134–141. 10.1080/09064719309411231

[CR44] He Z-H, Fujiki M, Kohorn BD (1996) A Cell Wall-associated, Receptor-like Protein Kinase. J Biol Chem 271:19789–19793. 10.1074/jbc.271.33.19789 8702686

[CR42] He Z, He D, Kohorn BD (1998) Requirement for the induced expression of a cell wall associated receptor kinase for survival during the pathogen response. Plant J 14:55–63. 10.1046/j.1365-313X.1998.00092.x 9681026

[CR43] He Z-H, Cheeseman I, He D, Kohorn BD (1999) A cluster of five cell wall-associated receptor kinase genes, *Wak1–5*, are expressed in specific organs of *Arabidopsis*. Plant Mol Biol 39:1189–1196 10.1023/a:1006197318246 10380805

[CR45] Hou X, Tong H, Selby J et al (2005) Involvement of a Cell Wall-Associated Kinase, *WAKL4*, in Arabidopsis Mineral Responses. Plant Physiol 139:1704–1716. 10.1104/pp.105.066910 PMC1310553 16286448

[CR46] Hu J, Chen G, Xu K, Wang J (2022) Cadmium in Cereal Crops: Uptake and Transport Mechanisms and Minimizing Strategies. J Agric Food Chem 70:5961–5974. 10.1021/acs.jafc.1c07896 35576456

[CR47] Huang M, Liu X, Zhou Y et al (2019) BLINK: a package for the next level of genome-wide association studies with both individuals and markers in the millions. 10.1093/gigascience/giy154. GigaScience 8:PMC6365300 30535326

[CR48] Hussain W, Campbell MT, Jarquin D et al (2020) Variance heterogeneity genome-wide mapping for cadmium in bread wheat reveals novel genomic loci and epistatic interactions. Plant Genome 13. 10.1002/tpg2.20011 PMC12807127 33016629

[CR49] Ishikawa S, Abe T, Kuramata M et al (2010) A major quantitative trait locus for increasing cadmium-specific concentration in rice grain is located on the short arm of chromosome 7. J Exp Bot 61:923–934. 10.1093/jxb/erp360 PMC2814118 20022924

[CR50] Kamal N, Tsardakas Renhuldt N, Bentzer J et al (2022) The mosaic oat genome gives insights into a uniquely healthy cereal crop. Nature 606:113–119. 10.1038/s41586-022-04732-y PMC9159951 35585233

[CR51] Kintlová M, Vrána J, Hobza R et al (2021) Transcriptome Response to Cadmium Exposure in Barley (*Hordeum vulgare* L). Front Plant Sci 12:629089. 10.3389/fpls.2021.629089 PMC8321094 34335638

[CR52] Kohorn BD, Kobayashi M, Johansen S et al (2006) An Arabidopsis cell wall-associated kinase required for invertase activity and cell growth. Plant J 46:307–316. 10.1111/j.1365-313X.2006.02695.x 16623892

[CR53] Li J, Zhang M, Sun J et al (2020) Heavy Metal Stress-Associated Proteins in Rice and Arabidopsis: Genome-Wide Identification, Phylogenetics, Duplication, and Expression Profiles Analysis. Front Genet 11:477. 10.3389/fgene.2020.00477 PMC7225358 32457808

[CR54] LiLin-Yin (2015) CMplot: Circle Manhattan Plot. 4.5.1

[CR55] Lipka AE, Tian F, Wang Q et al (2012) GAPIT: genome association and prediction integrated tool. Bioinformatics 28:2397–2399. 10.1093/bioinformatics/bts444 22796960

[CR56] Liu K, Lv J, He W et al (2015) Major factors influencing cadmium uptake from the soil into wheat plants. Ecotoxicol Environ Saf 113:207–213. 10.1016/j.ecoenv.2014.12.005 25499054

[CR57] Liu X, Huang M, Fan B et al (2016) Iterative Usage of Fixed and Random Effect Models for Powerful and Efficient Genome-Wide Association Studies. PLOS Genet 12:e1005767. 10.1371/journal.pgen.1005767 PMC4734661 26828793

[CR58] Ma B-L, Zheng Z, Ren C (2021) Chapter 6 - Oat. Editor(s): Victor O. Sadras and Daniel F. Calderini, Crop Physiology Case Histories for Major Crops, Academic Press, pp 222–248. 10.1016/B978-0-12-819194-1.00006-2

[CR59] Ma X, Yang H, Bu Y et al (2023) Genome-wide identification of PLATZ genes related to cadmium tolerance in *Populus trichocarpa* and characterization of the role of PtPLATZ3 in phytoremediation of cadmium. Int J Biol Macromol 228:732–743. 10.1016/j.ijbiomac.2022.12.176 36563811

[CR60] Mei S, Lin K, Williams DV et al (2022) Cadmium Accumulation in Cereal Crops and Tobacco: A Review. Agronomy 12:1952. 10.3390/agronomy12081952

[CR61] Oliveira G, Zimmer CM, Federizzi LC, Pacheco MT (2025) Genome-wide association mapping for thousand kernel weight in oat germplasm adapted to the subtropic. Cereal Res Commun. 10.1007/s42976-025-00659-y

[CR62] Patterson N, Price AL, Reich D (2006) Population Structure and Eigenanalysis. PLoS Genet 2:e190. 10.1371/journal.pgen.0020190 PMC1713260 17194218

[CR63] Peng Y, Yan H, Guo L et al (2022) Reference genome assemblies reveal the origin and evolution of allohexaploid oat. Nat Genet 54:1248–1258. 10.1038/s41588-022-01127-7 PMC9355876 35851189

[CR64] Peterson DM (2001) Oat Antioxidants. J Cereal Sci 33:115–129. 10.1006/jcrs.2000.0349

[CR65] Polley A, Plieske J, Grafarend-Belau E et al (2023) A new 7K SNP genotyping array for oat. Oat Newsl 60, No 4, pp 1-3.

[CR66] Pritchard JK, Stephens M, Donnelly P (2000) Inference of Population Structure Using Multilocus Genotype Data. Genetics 155:945–959. 10.1093/genetics/155.2.945 PMC1461096 10835412

[CR67] Rizwan M, Ali S, Abbas T et al (2016) Cadmium minimization in wheat: A critical review. Ecotoxicol Environ Saf 130:43–53. 10.1016/j.ecoenv.2016.04.001 27062345

[CR68] Roy SK, Cho S-W, Kwon SJ et al (2016) Morpho-Physiological and Proteome Level Responses to Cadmium Stress in Sorghum. PLoS ONE 11:e0150431. 10.1371/journal.pone.0150431 PMC4769174 26919231

[CR69] Safdar LB, Almas F, Sarfraz S et al (2020) Genome-wide association study identifies five new cadmium uptake loci in wheat. Plant Genome 13. 10.1002/tpg2.20030 PMC12807261 33016603

[CR70] Salt DE, Wagner GJ (1993) Cadmium transport across tonoplast of vesicles from oat roots. Evidence for a Cd2+/H+ antiport activity. J Biol Chem 268:12297–12302. 10.1016/S0021-9258(18)31388-7 8509367

[CR71] Sasaki A, Yamaji N, Ma JF (2014) Overexpression of *OsHMA3* enhances Cd tolerance and expression of Zn transporter genes in rice. J Exp Bot 65:6013–6021. 10.1093/jxb/eru340 PMC4203134 25151617

[CR72] Segura V, Vilhjálmsson BJ, Platt A et al (2012) An efficient multi-locus mixed-model approach for genome-wide association studies in structured populations. Nat Genet 44:825–830. 10.1038/ng.2314 PMC3386481 22706313

[CR73] Sferra G, Fantozzi D, Scippa GS, Trupiano D (2023) Key Pathways and Genes of *Arabidopsis thaliana* and *Arabidopsis halleri* Roots under Cadmium Stress Responses: Differences and Similarities. Plants 12:1793. 10.3390/plants12091793 PMC10180823 37176850

[CR74] Sivaguru M, Ezaki B, He Z-H et al (2003) Aluminum-Induced Gene Expression and Protein Localization of a Cell Wall-Associated Receptor Kinase in Arabidopsis. Plant Physiol 132:2256–2266. 10.1104/pp.103.022129 PMC181309 12913180

[CR75] Suzuki N, Yamaguchi Y, Koizumi N, Sano H (2002) Functional characterization of a heavy metal binding protein CdI19 from *Arabidopsis*. Plant J 32:165–173. 10.1046/j.1365-313X.2002.01412.x 12383082

[CR76] Tang B, Luo M, Zhang Y et al (2021a) Natural variations in the P-type ATPase heavy metal transporter gene *ZmHMA3* control cadmium accumulation in maize grains. J Exp Bot 72:6230–6246. 10.1093/jxb/erab254 34235535

[CR77] Tang L, Dong J, Tan L et al (2021b) Overexpression of *OsLCT2*, a Low-Affinity Cation Transporter Gene, Reduces Cadmium Accumulation in Shoots and Grains of Rice. Rice 14:89. 10.1186/s12284-021-00530-8 PMC8542528 34693475

[CR78] Tanhuanpää P, Kalendar R, Schulman AH, Kiviharju E (2007) A major gene for grain cadmium accumulation in oat (*Avena sativa* L). Genome 50:588–594. 10.1139/G07-036 17632580

[CR79] Tao J, Lu L (2022) Advances in Genes-Encoding Transporters for Cadmium Uptake, Translocation, and Accumulation in Plants. Toxics 10:411. 10.3390/toxics10080411 PMC9332107 35893843

[CR80] Tapola N, Karvonen H, Niskanen L et al (2005) Glycemic responses of oat bran products in type 2 diabetic patients. Nutr Metab Cardiovasc Dis 15:255–261. 10.1016/j.numecd.2004.09.003 16054549

[CR81] Tinker NA, Wight CP, Bekele WA et al (2022) Genome analysis in *Avena sativa* reveals hidden breeding barriers and opportunities for oat improvement. Commun Biol 5:474. 10.1038/s42003-022-03256-5 PMC9117302 35585176

[CR82] Ubert IP, Zimmer CM, Pellizzaro K et al (2017) Genetics and molecular mapping of the naked grains in hexaploid oat. Euphytica 213:41. 10.1007/s10681-017-1836-1

[CR83] Ueno D, Kono I, Yokosho K et al (2009) A major quantitative trait locus controlling cadmium translocation in rice (*Oryza sativa*). New Phytol 182:644–653. 10.1111/j.1469-8137.2009.02784.x 19309445

[CR84] Ueno D, Yamaji N, Kono I et al (2010) Gene limiting cadmium accumulation in rice. Proc Natl Acad Sci 107:16500–16505. 10.1073/pnas.1005396107 PMC2944702 20823253

[CR85] Uraguchi S, Kamiya T, Sakamoto T et al (2011) Low-affinity cation transporter (*OsLCT1*) regulates cadmium transport into rice grains. Proc Natl Acad Sci 108:20959–20964. 10.1073/pnas.1116531109 PMC3248505 22160725

[CR86] VanRaden PM (2008) Efficient Methods to Compute Genomic Predictions. J Dairy Sci 91:4414–4423. 10.3168/jds.2007-0980 18946147

[CR87] Verica JA, He Z-H (2002) The Cell Wall-Associated Kinase (*WAK*) and *WAK*-Like Kinase Gene Family. Plant Physiol 129:455–459. 10.1104/pp.011028 PMC1540232 12068092

[CR88] Wagner TA, Kohorn BD (2001) Wall-Associated Kinases Are Expressed throughout Plant Development and Are Required for Cell Expansion. Plant Cell 13:303–318. 10.1105/tpc.13.2.303 PMC102244 11226187

[CR89] Wang J, Zhang Z (2021) GAPIT Version 3: Boosting Power and Accuracy for Genomic Association and Prediction. Genomics Proteom Bioinf 19:629–640. 10.1016/j.gpb.2021.08.005 PMC9121400 34492338

[CR90] Wei T, Simko V (2017) R package corrplot. Visualization of a Correlation Matrix

[CR91] Wei Y, Peng X, Wang X, Wang C (2023) The heavy metal-associated isoprenylated plant protein (HIPP) gene family plays a crucial role in cadmium resistance and accumulation in the tea plant (*Camellia sinensis* L). Ecotoxicol Environ Saf 260:115077. 10.1016/j.ecoenv.2023.115077 37257351

[CR92] Welch RM, Norvell WA (1999) Mechanisms of Cadmium Uptake, Translocation and Deposition in Plants. In: McLaughlin MJ, Singh BR (eds) Cadmium in Soils and Plants. Springer Netherlands, Dordrecht, pp 125–150

[CR93] Wu D, Sato K, Ma JF (2015) Genome-wide association mapping of cadmium accumulation in different organs of barley. New Phytol 208:817–829. 10.1111/nph.13512 26061418

[CR94] Xia Y, Yin S, Zhang K et al (2018) *OsWAK11*, a rice wall-associated kinase, regulates Cu detoxification by alteration the immobilization of Cu in cell walls. Environ Exp Bot 150:99–105. 10.1016/j.envexpbot.2018.03.005

[CR95] Yan H, Deng D, Zhou P et al (2023) Dissecting the genetic basis of grain weight and size in common oat by genome-wide association study. J Cereal Sci 114:103811. 10.1016/j.jcs.2023.103811

[CR96] Yang X, Baligar VC, Martens DC, Clark RB (1995) Influx, transport, and accumulation of cadmium in plant species grown at different Cd2 + activities. J Environ Sci Health Part B 30:569–583. 10.1080/03601239509372954

[CR97] Yin X, Hou X (2017) Role of *OsWAK124*, a rice wall-associated kinase, in response to environmental heavy metal stresses. Pak J Bot 49:1255–1261

[CR100] Zhang Z, Ersoz E, Lai C-Q et al (2010) Mixed linear model approach adapted for genome-wide association studies. Nat Genet 42:355–360. 10.1038/ng.546 PMC2931336 20208535

[CR98] Zhang H, Zhang X, Liu J et al (2020a) Characterization of the Heavy-Metal-Associated Isoprenylated Plant Protein (HIPP) Gene Family from Triticeae Species. Int J Mol Sci 21:6191. 10.3390/ijms21176191 PMC7504674 32867204

[CR99] Zhang L, Gao C, Chen C et al (2020b) Overexpression of Rice *OsHMA3* in Wheat Greatly Decreases Cadmium Accumulation in Wheat Grains. Environ Sci Technol 54:10100–10108. 10.1021/acs.est.0c02877 32697086

[CR101] Zhao X, Luo L, Cao Y et al (2018) Genome-wide association analysis and QTL mapping reveal the genetic control of cadmium accumulation in maize leaf. BMC Genomics 19:91. 10.1186/s12864-017-4395-x PMC5785805 29370753

[CR102] Zimmer CM, McNish IG, Esvelt Klos K et al (2021) Genome-wide association mapping for kernel shape and its association with β‐glucan content in oats. Crop Sci 61:3986–3999. 10.1002/csc2.20605

