# Supplementary material for: Two major QTL for Cadmium accumulation in oat (Avena sativa L.) grains
Source: Theor Appl Genet. 2026 Nov 11;139(12):330. doi: 10.1007/s00122-026-05427-y (PMC13755090; doi:10.1007/s00122-026-05427-y)
Supplement: Supplementary file 1 [file 122_2026_5427_MOESM1_ESM.pdf]

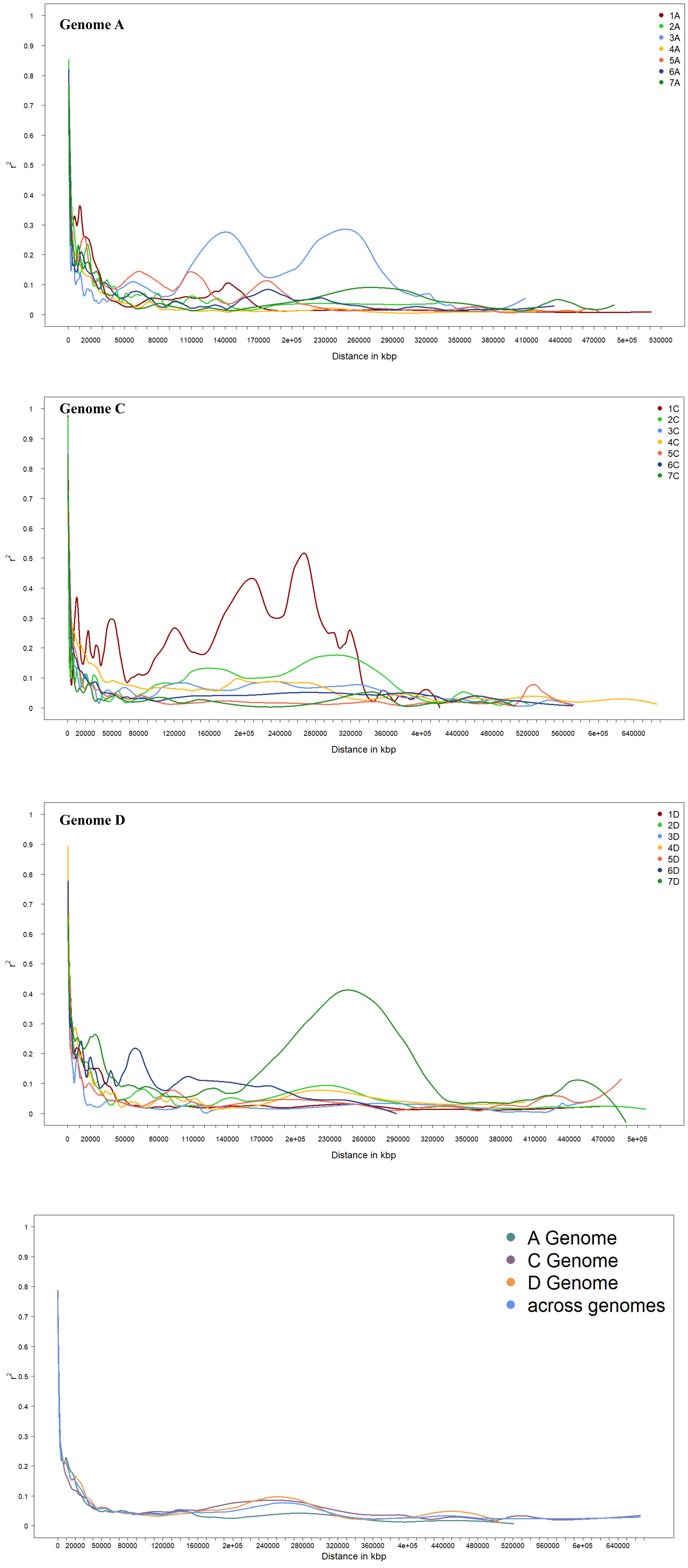

Supplementary Figure 2 Linkage disequilibrium (LD) decay of a population 596 Nordic spring oat breeding lines calculated for each sub-genome and respective chromosomes.
