# Supplementary material for: Two major QTL for Cadmium accumulation in oat (Avena sativa L.) grains
Source: Theor Appl Genet. 2026 Nov 11;139(12):330. doi: 10.1007/s00122-026-05427-y (PMC13755090; doi:10.1007/s00122-026-05427-y)
Supplement: Supplementary file 2 [file 122_2026_5427_MOESM2_ESM.pdf]

a)

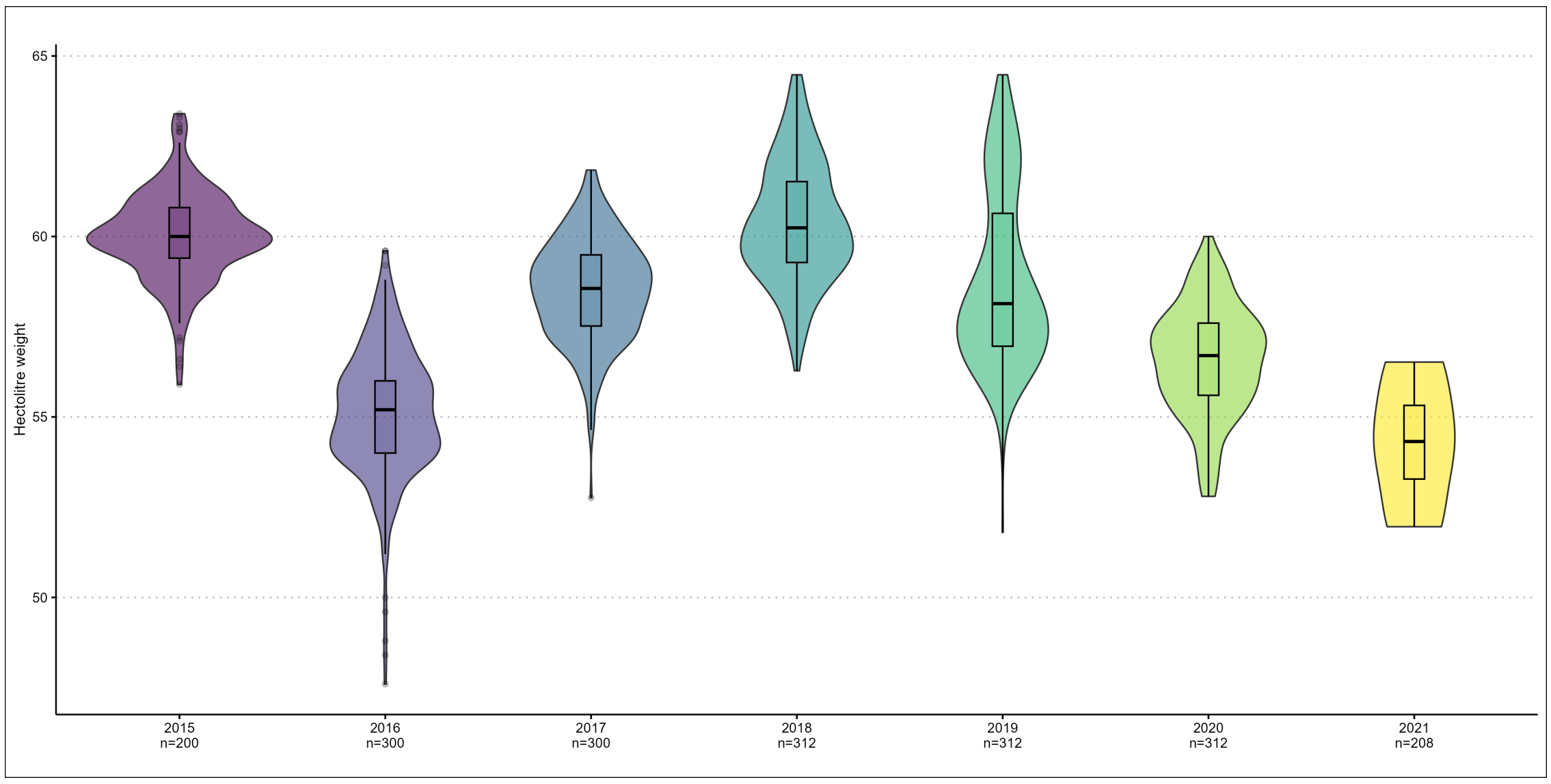

b)

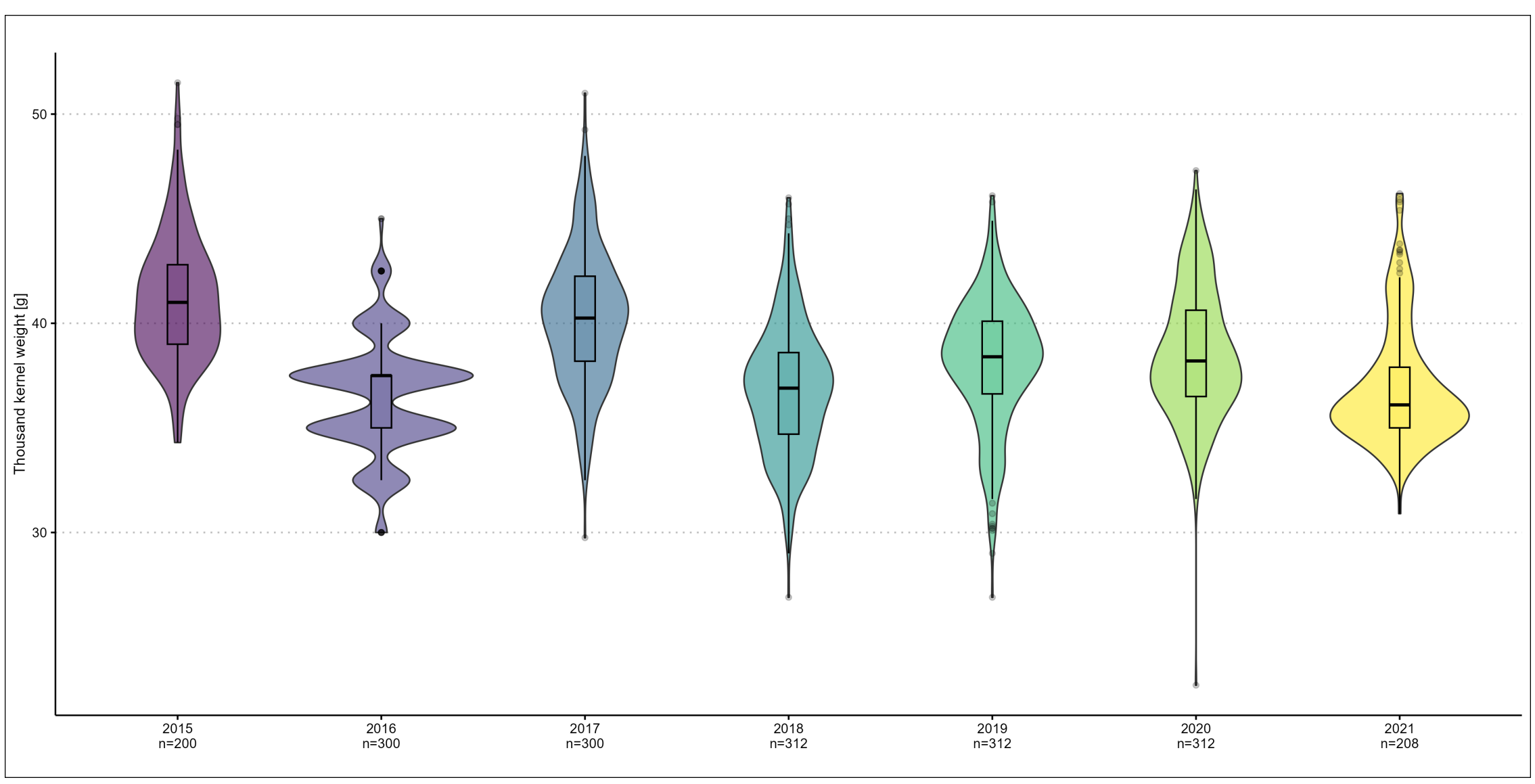

c)

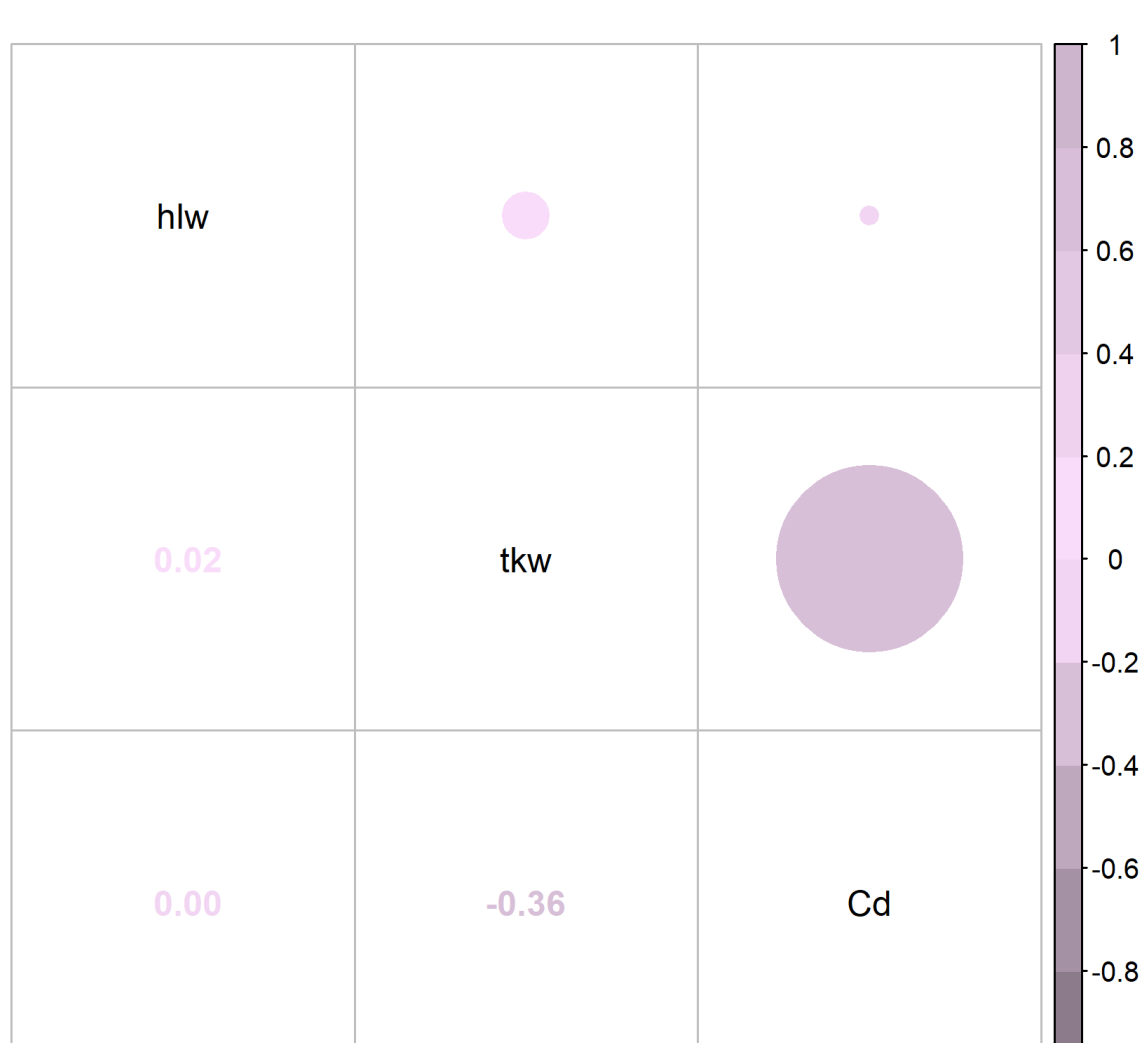

Supplementary Figure 1 Violin plots for spring oat breeding lines grown under field conditions in Svalöv, Southern Sweden, analysed for a) hectolitre weight and b) thousand kernel weight. Box plots represent the minimum, first quartile, median, third quartile, and maximum. Numbers underneath each year represent the number of lines tested in the respective year, including the three check cultivars, 'Belinda', 'Galant', and 'Nike'. c) Pearson correlation between the three traits.
