# Supplementary material for: Two major QTL for Cadmium accumulation in oat (Avena sativa L.) grains
Source: Theor Appl Genet. 2026 Nov 11;139(12):330. doi: 10.1007/s00122-026-05427-y (PMC13755090; doi:10.1007/s00122-026-05427-y)
Supplement: Supplementary file 3 [file 122_2026_5427_MOESM3_ESM.pdf]

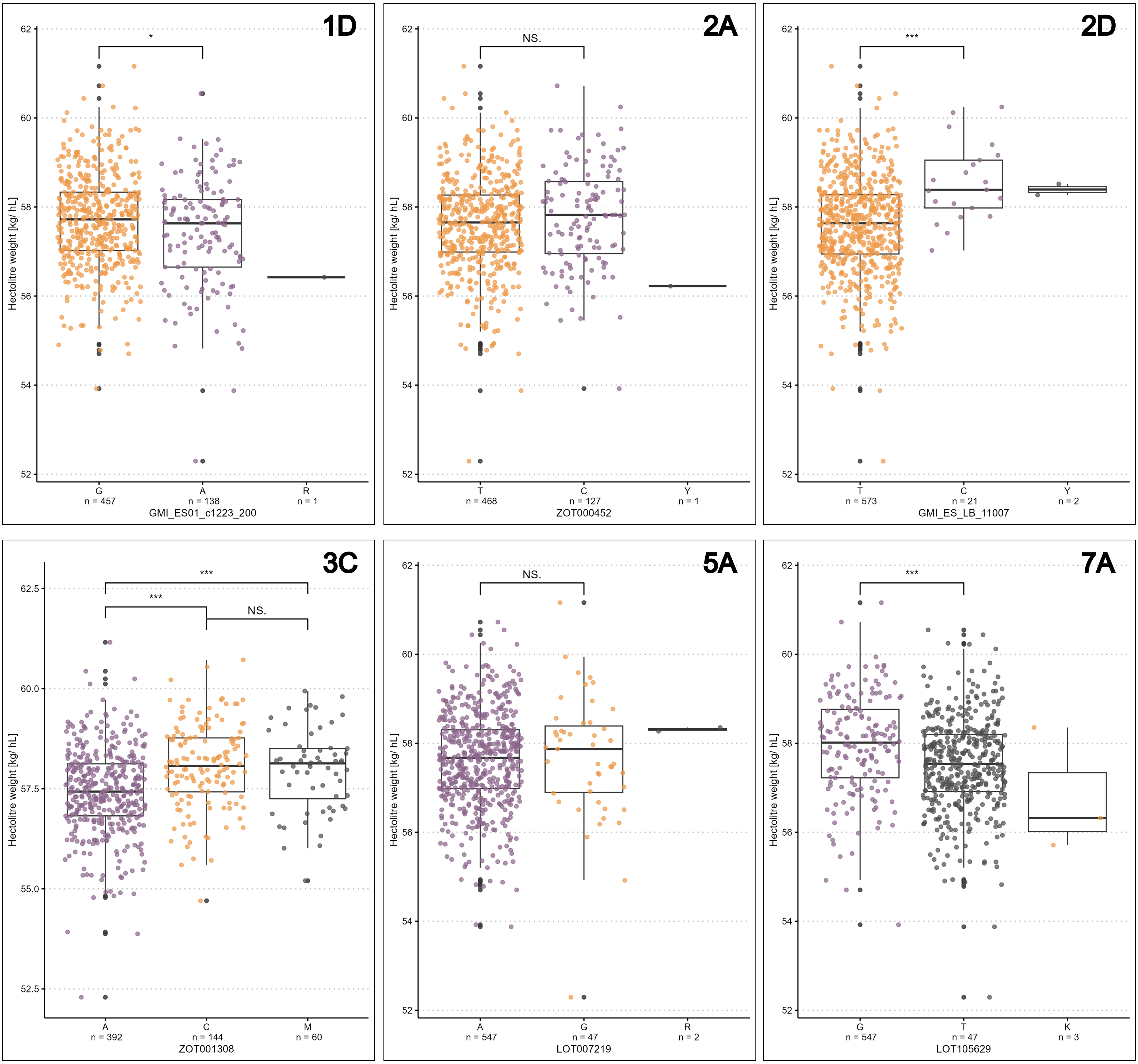

Supplementary Figure 4 Allele effects on hectolitre weight (hlw) for MTAs identified in a spring oat breeding population located on six chromosomes. Orange, lilac and light grey dots represent values of hlw for each individual line. dark grey dots represent outliers. Asterisk represent significance thresholds of the Wilcox test between groups with '*' - $p < 0.05$, '**' - $p < 0.01$, '***' - $p < 0.001$, NS - not significant, n indicates the number of observations per group.
