# Supplementary material for: Two major QTL for Cadmium accumulation in oat (Avena sativa L.) grains
Source: Theor Appl Genet. 2026 Nov 11;139(12):330. doi: 10.1007/s00122-026-05427-y (PMC13755090; doi:10.1007/s00122-026-05427-y)
Supplement: Supplementary file 5 [file 122_2026_5427_MOESM5_ESM.pdf]

Cadmium Grain Content

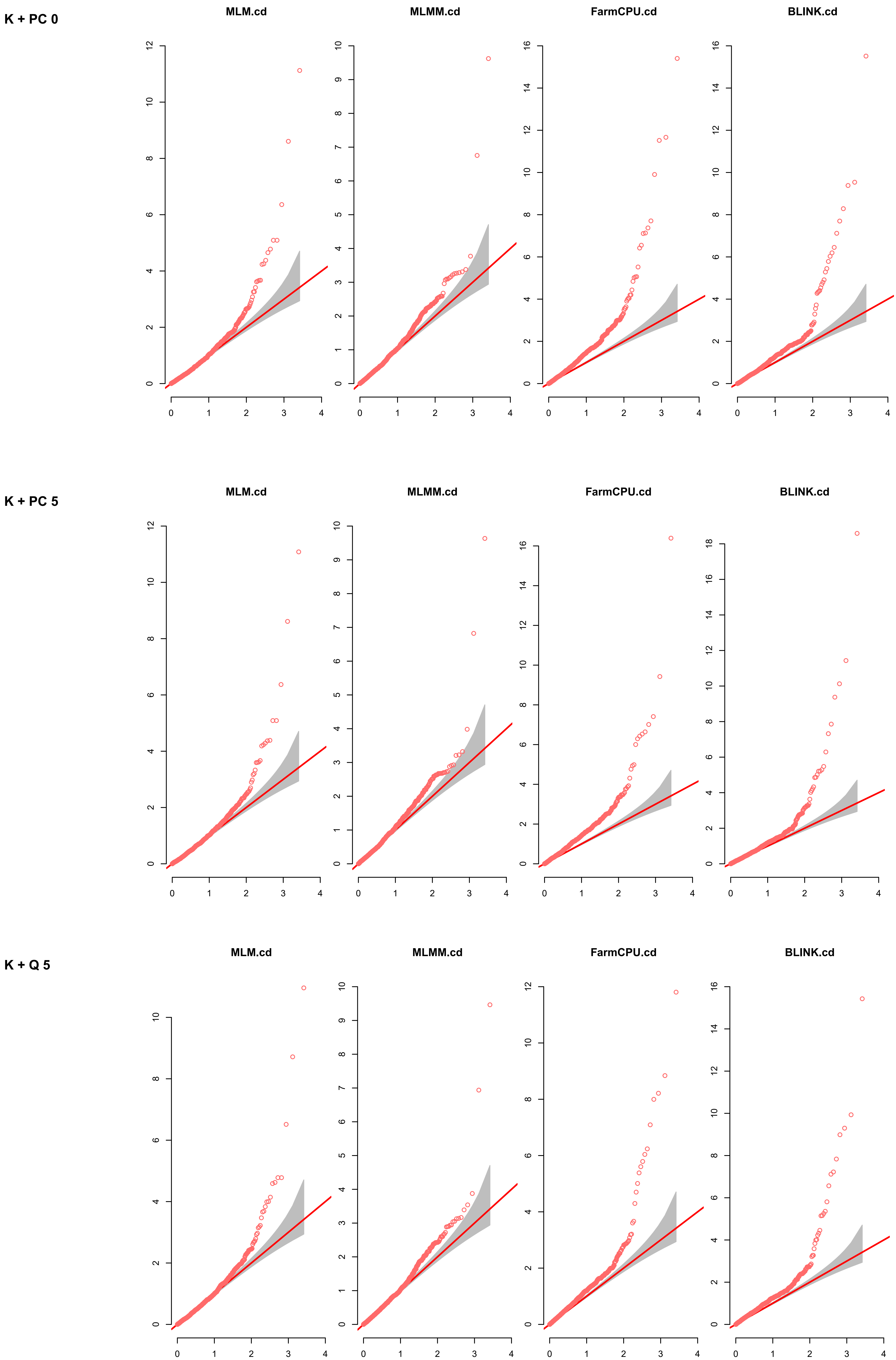

Hectolitre Weight

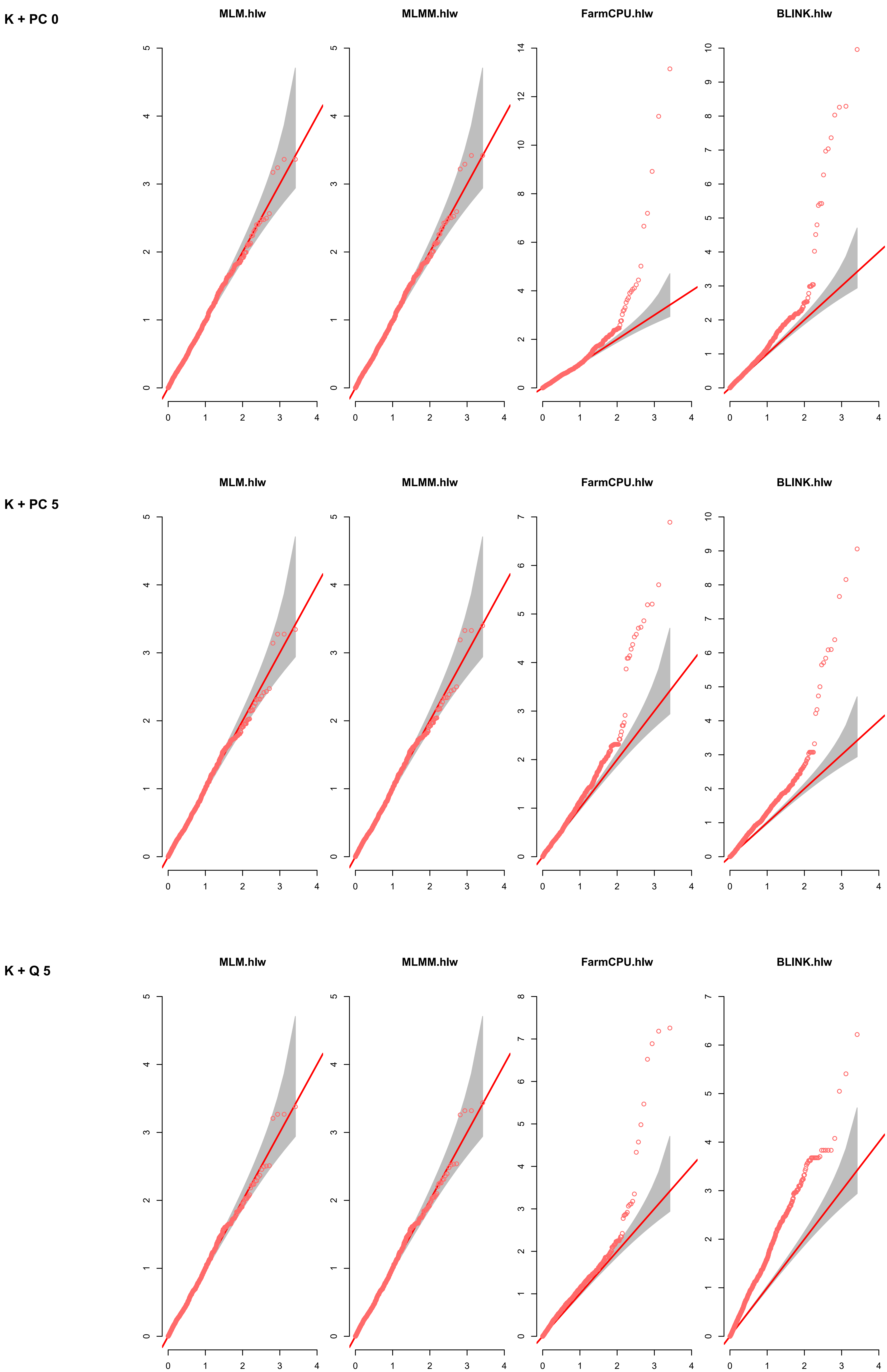

Thousand Kernel Weight

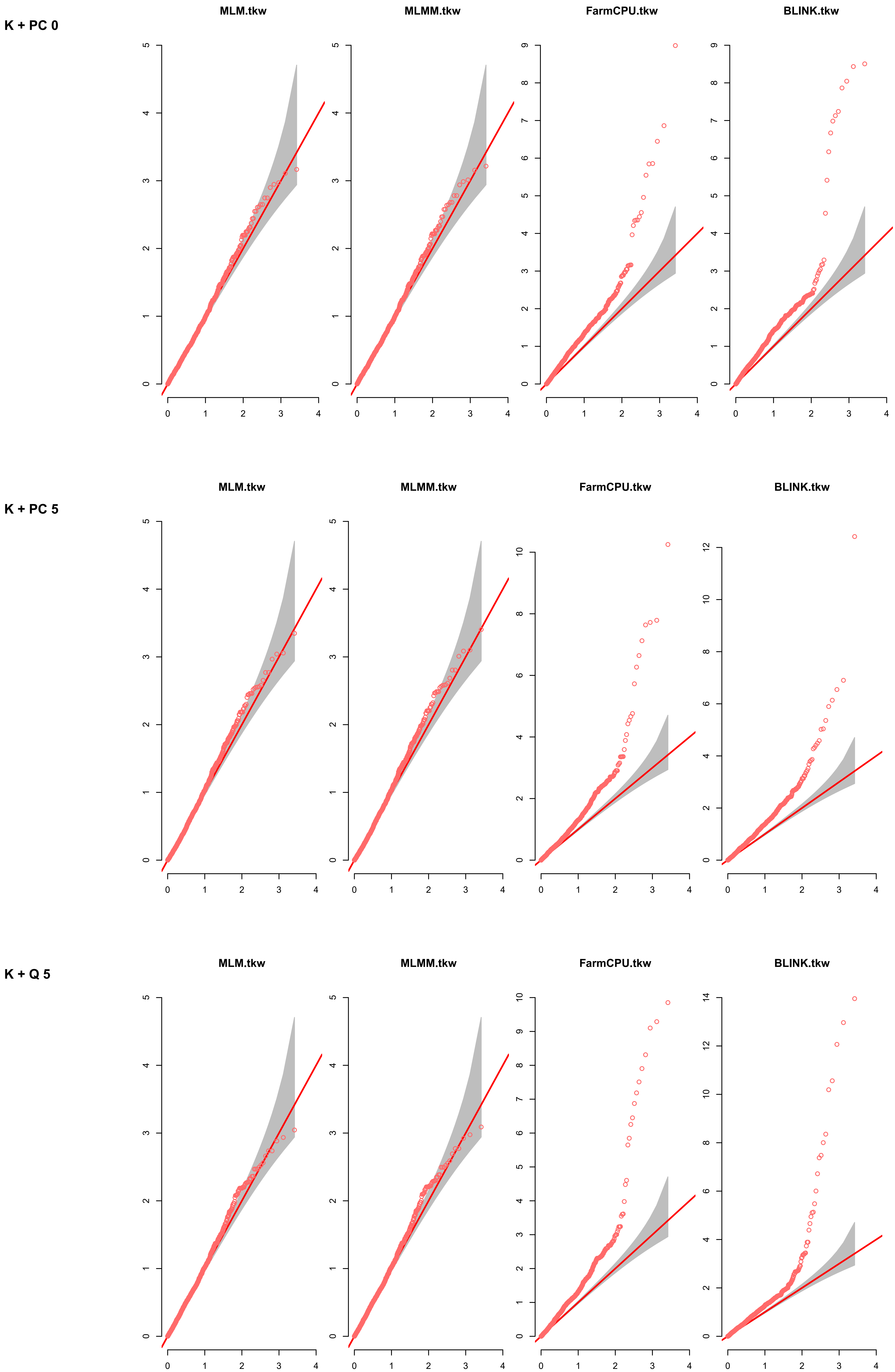

Supplementary Figure 3 QQ plots for genome wide association studies for cadmium content, hectolitre weight and thousand kernel weight in a spring out breeding population.
