# Supplementary material for: Two major QTL for Cadmium accumulation in oat (Avena sativa L.) grains
Source: Theor Appl Genet. 2026 Nov 11;139(12):330. doi: 10.1007/s00122-026-05427-y (PMC13755090; doi:10.1007/s00122-026-05427-y)
Supplement: Supplementary file 6 [file 122_2026_5427_MOESM6_ESM.pdf]

a)
b)

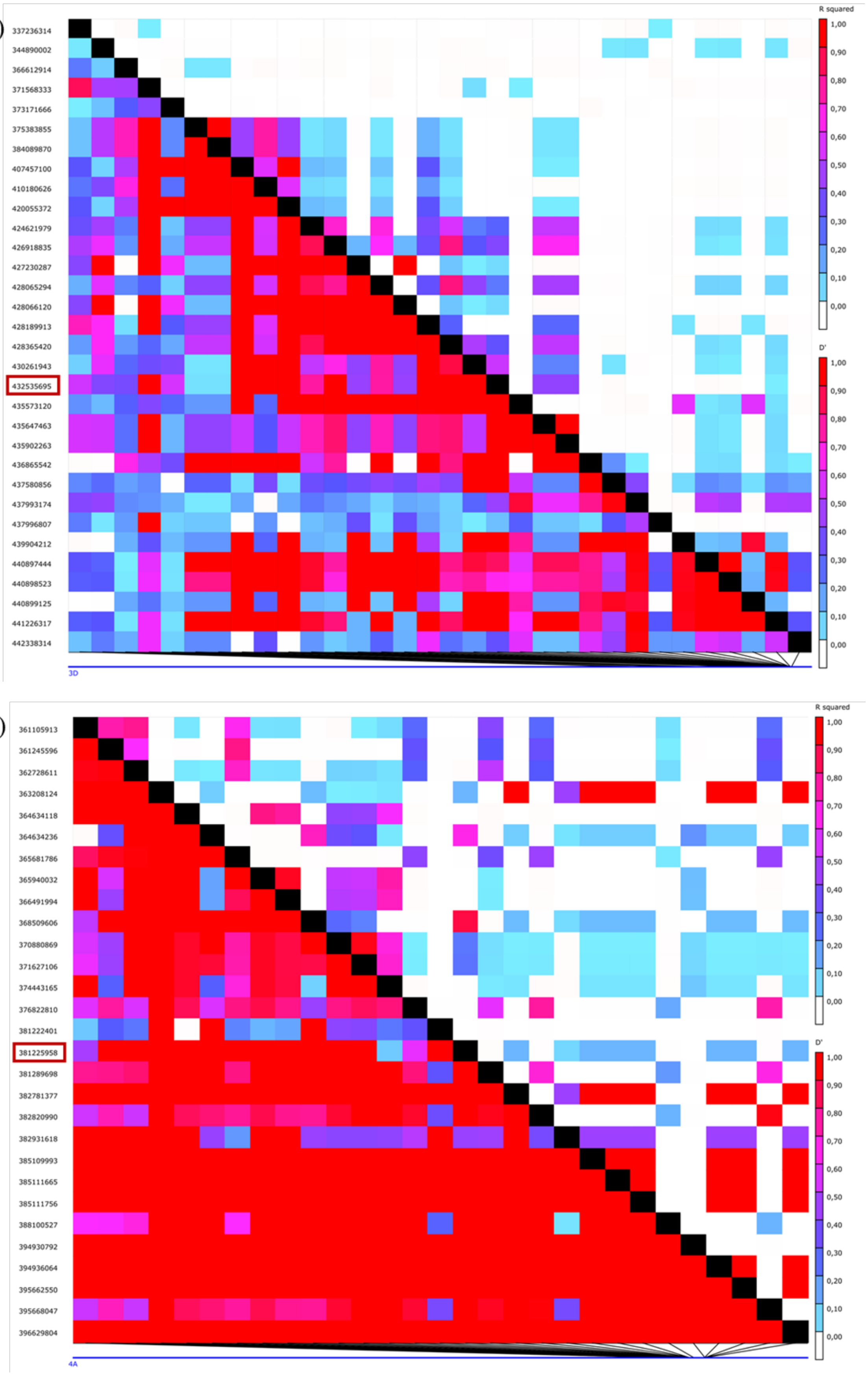

Supplementary Figure 6 LD heatmaps of regions surrounding the significant SNPs on a) chromosome 3D and b) chromosome 4A. On the left the physical positions of the SNPs are shown, red boxes indicate the significant SNP. The upper triangles show the r² value, the lower the D'-value.
