# Supplementary material for: Two major QTL for Cadmium accumulation in oat (Avena sativa L.) grains
Source: Theor Appl Genet. 2026 Nov 11;139(12):330. doi: 10.1007/s00122-026-05427-y (PMC13755090; doi:10.1007/s00122-026-05427-y)
Supplement: Supplementary file 7 [file 122_2026_5427_MOESM7_ESM.pdf]

a)

```
                      1620      1630      1640      1650      1660      1670      1680
                  ....|....|....|....|....|....|....|....|....|....|....|....|....|....|
SOA369     1610   GGGGGGGCAAAATACAATATTTGATGTCTTGGGGGGGCAAAATACACAAAAAATTCCAACCAAAGCTATC
SOA372     1608   GGGGGGGCAAAATACAATATTTGATGTCTTGGGGGGGCAAAATACACAAAAAATTCCAACCAAAGCTATC
Active     1610   GGGGGGGCAAAATACAATATTTGATGTCTTGGGGGGGCAAAATACACAAAAAATTCCAACCAAAGCTATC
Hera       1611   GGGGGGGCAAAATACAATATTTGATGTCTTGGGGGGGCAAAATACACAAAAAATTCCAACCAAAGCTATC
Welinder   1606   GGGGGGGCAAAATACAATATTTGATGTCTTGGGGGGGCAAAATACACAAAAAATTCCAACCAAAGCTATC
SOA026     1611   GGGGGGGCAAAATACAATATTTGATGTCTTGGGGGGGCAAAATACACAAAAAATTCCAACCAAAGCTATC
SOA599     1610   GGGGGGGCAAAATACAATATTTGATGTCTTGGGGGGGCAAAATACACAAAAAATTCCAACCAAAGCTATC
SOA608     1610   GGGGGGGCAAAATACAATATTTGATGTCTTGGGGGGGCAAAATACACAAAAAATTCCAACCAAAGCTATC

                      1690      1700      1710      1720      1730      1740      1750
                  ....|....|....|....|....|....|....|....|....|....|....|....|....|....|
SOA369     1680   TCAGATTTTTTTCTTCAAGCATGTGCTCAATCTTGGGGGG--GGAGGGGGGGGGGGGGGGGTGGCCCCT
SOA372     1678   TCAGATTTTTTTCTTCAAGCATGTGCTCAATCTTGGGGGG--AGGGGGGGAGGGGGGGGGGGTGGCCCCT
Active     1680   TCAGATTTTTTTCTTCAAGCATGTGCTCAATCTTGGGGGGGGGAGGGGGGGAGGGGGGGGGGTGGCCCCT
Hera       1681   TCAGATTTTTTTCTTCAAGCATGTGCTCAATCTTGGGGGGG-GAGGGGGGAGGGGGGGGGGGTGGCCCCT
Welinder   1676   TCAGATTTTTTTCTTCAAGCATGTGCTCAATCTTGGGGGGG-GAGGGGGGAGGGGGGGGGGGTGGCCCCT
SOA026     1681   TCAGATTTTTTTCTTCAAGCATGTGCTCAATCTTGGGGGGG-GAGGGGGGAGGGGGGGGGGGTGGCCCCT
SOA599     1680   TCAGATTTTTTTCTTCAAGCATGTGCTCAATCTTGGGGGGGGAGGGGGGGAGGGGGGGGGGGTGGCCCCT
SOA608     1680   TCAGATTTTTTTCTTCAAGCATGTGCTCAATCTTGGGGGG--GGGGGGGGAGGGGGGGGGGGTGGCCCCT

                      1760      1770      1780      1790      1800      1810      1820
                  ....|....|....|....|....|....|....|....|....|....|....|....|....|....|
SOA369     1748   GCTGGCCTGCACTAAGCTCCGCCACTGACGCCGATGACAGCCTCATCGCCAACTTCCATGATAGCTTTTT
SOA372     1746   GCTGGCCTGCACTAAGCTCCGCCACTGACGCCGATGACAGCCTCATCGCCAACTTCCATGATAGCTTTTT
Active     1750   GCTGGCCTGCACTAAGCTCCGCCACTGACGCCGATGACAGCCTCATCGCCAACTTCCATGATAGCTTTTT
Hera       1750   GCTGGCCTGCACTAAGCTCCGCCACTGACGCCGATGACAGCCTCATCGCCAACTTCCATGATAGCTTTTT
Welinder   1745   GCTGGCCTGCACTAAGCTCCGCCACTGACGCCGATGACAGCCTCATCGCCAACTTCCATGATAGCTTTTT
SOA026     1750   GCTGGCCTGCACTAAGCTCCGCCACTGACGCCGATGACAGCCTCATCGCCAACTTCCATGATAGCTTTTT
SOA599     1750   GCTGGCCTGCACTAAGCTCCGCCACTGACGCCGATGACAGCCTCATCGCCAACTTCCATGATAGCTTTTT
SOA608     1748   GCTGGCCTGCACTAAGCTCCGCCACTGACGCCGATGACAGCCTCATCGCCAACTTCCATGATAGCTTTTT
```

b)

```
                      3650      3660      3670      3680      3690      3700      3710
                  ....|....|....|....|....|....|....|....|....|....|....|....|....|....|
SOA369     3640   AAGAATTAGAGGGGCTAGAATCGTGCCTTGGATAAACTGGCTAGGCAAACATGAACGAACTCAACGAAGT
SOA372     3637   AAGAATTAGAGGGGCTAGAATCGTGCCTTGGATAAACTGGCTAGGCAAACATGAACGAACTCAACGAAGT
Active     3595   AAGAATTAGAGGGGCTAGAATCGTGCCTTGGATAAACTGGCTAGGCAAACATGAACGAACTCAACGAAGT
Hera       3499   AAGAATTAGAGGGGCTAGAATCGTGCCTTGGATAAACTGGCTAGGCAAACATGAACGAACTCAACGAAGT
Welinder   3641   AAGAATTAGAGGGGCTAGAATCGTGCCTTGGATAAACTGGCTAGGCAAACATGAACGAACTCAACGAAGT
SOA599     3641   AAGAATTAGAGGGGCTAGAATCGTGCCTTGGATAAACTGGCTAGGCAAACATGAACGAACTCAACGAAGT
SOA608     3641   AAGAATTAGAGGGGCTAGAATCGTGCCTTGGATAAACTGGCTAGGCAAACATGAACGAACTCAACGAAGT

                      3720      3730      3740      3750      3760      3770      3780
                  ....|....|....|....|....|....|....|....|....|....|....|....|....|....|
SOA369     3710   GCTGCCGTGTTGGAGTGGAGCGCCTGCTGCCGGCCTGCTACTGGGATGCTGCACCGCCAGCCACGGGGGA
SOA372     3707   GCTGCCGTGTTGGAGTGGAGCGCCTGCTGCCGGCCTGCTACTGGGATGCTGCACCGCCAGCCACGGGGGA
Active     3665   GCTGCCGTGTTGGAGTGGAGCGCCTGCTGCCGGCCTGCTACTGGGATGCTGCACCAACAGCCACGGGGGA
Hera       3569   GCTGCCGTGTTGGAGTGGAGCGCCTGCTGCCGGCCTGCTACTGGGATGCTGCACCGCCAGCCACGGGGGA
Welinder   3711   GCTGCCGTGTTGGAGTGGAGCGCCTGCTGCCGGCCTGCTACTGGGATGCTGCACCGCCAGCCACGGGGGA
SOA599     3711   GCTGCCGTGTTGGAGTGGAGCGCCTGCTGCCGGCCTGCTACTGGGATGCTGCACCGCCAGCCACGGGGGA
SOA608     3711   GCTGCCGTGTTGGAGTGGAGCGCCTGCTGCCGGCCTGCTACTGGGATGCTGCACCGCCAGCCACGGGGGA

                      3790      3800      3810      3820      3830      3840      3850
                  ....|....|....|....|....|....|....|....|....|....|....|....|....|....|
SOA369     3780   AGCAACAGGGTGCCGCCGTCGCCGGGCCGACGGGGCGGGCGTCAGGGGGCGCGCCTACCTGACCTCGGCT
SOA372     3777   AGCAACAGGGTGCCGCCGTCGCCGGGCCGACGGGGCGGGCGTCAGGGGGCGCGCCTACCTGACCTCGGCT
Active     3735   AGCAACAGGGTGCCGCCGTCGCCGGGCCGACGGGGCGGGCGTCAGGGGGCGCGCCTACCTGACCTCGGCT
Hera       3639   AGCAACAGGGTGCCGCCGTCGCCGGGCCGACGGGGCGGGCGTCAGGGGGCGCGCCTACCTGACCTCGGCT
Welinder   3781   AGCAACAGGGTGCCGCCGTCGCCGGGCCGACGGGGCGGGCGTCAGGGGGCGCGCCTACCTGACCTCGGCT
SOA599     3781   AGCAACAGGGTGCCGCCGTCGCCGGGCCGACGGGGCGGGCGTCAGGGGGCGCGCCTACCTGACCTCGGCT
SOA608     3781   AGCAACAGGGTGCCGCCGTCGCCGGGCCGACGGGGCGGGCGTCAGGGGGCGCGCCTACCTGACCTCGGCT
```

Supplementary Figure 8 Gene sequence excerpts of a) the promotor region (2000 bp upstream) for oat gene AVESA.00010b.r2.3DG0562400 (413 bp) coding for a heavy metal associated protein and b) for oat gene AVESA.00010b.r2.3DG0565980 (430 bp) coding for a wall associated kinase protein, located on chromosome 3D for eight and seven spring oat

breeding lines, respectively. The first five lines were low in Cd grain accumulation, the last three (a) and two (b) high in Cd grain accumulation.
