# Supplementary material for: Two major QTL for Cadmium accumulation in oat (Avena sativa L.) grains
Source: Theor Appl Genet. 2026 Nov 11;139(12):330. doi: 10.1007/s00122-026-05427-y (PMC13755090; doi:10.1007/s00122-026-05427-y)
Supplement: Supplementary file 8 [file 122_2026_5427_MOESM8_ESM.pdf]

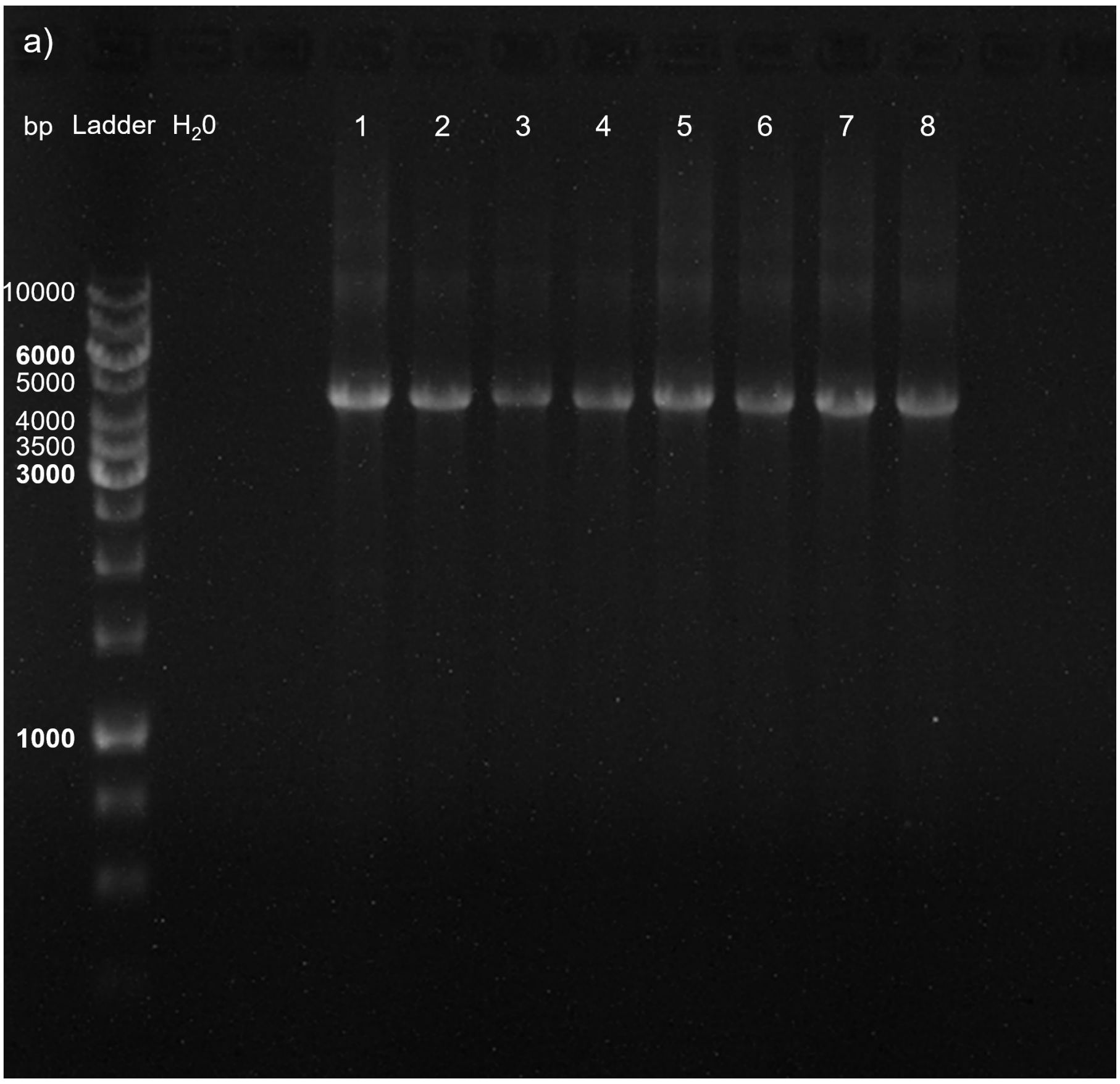
a)
bp Ladder H_20
1 2 3 4 5 6 7 8
10000
6000
5000
4000
3500
3000
1000

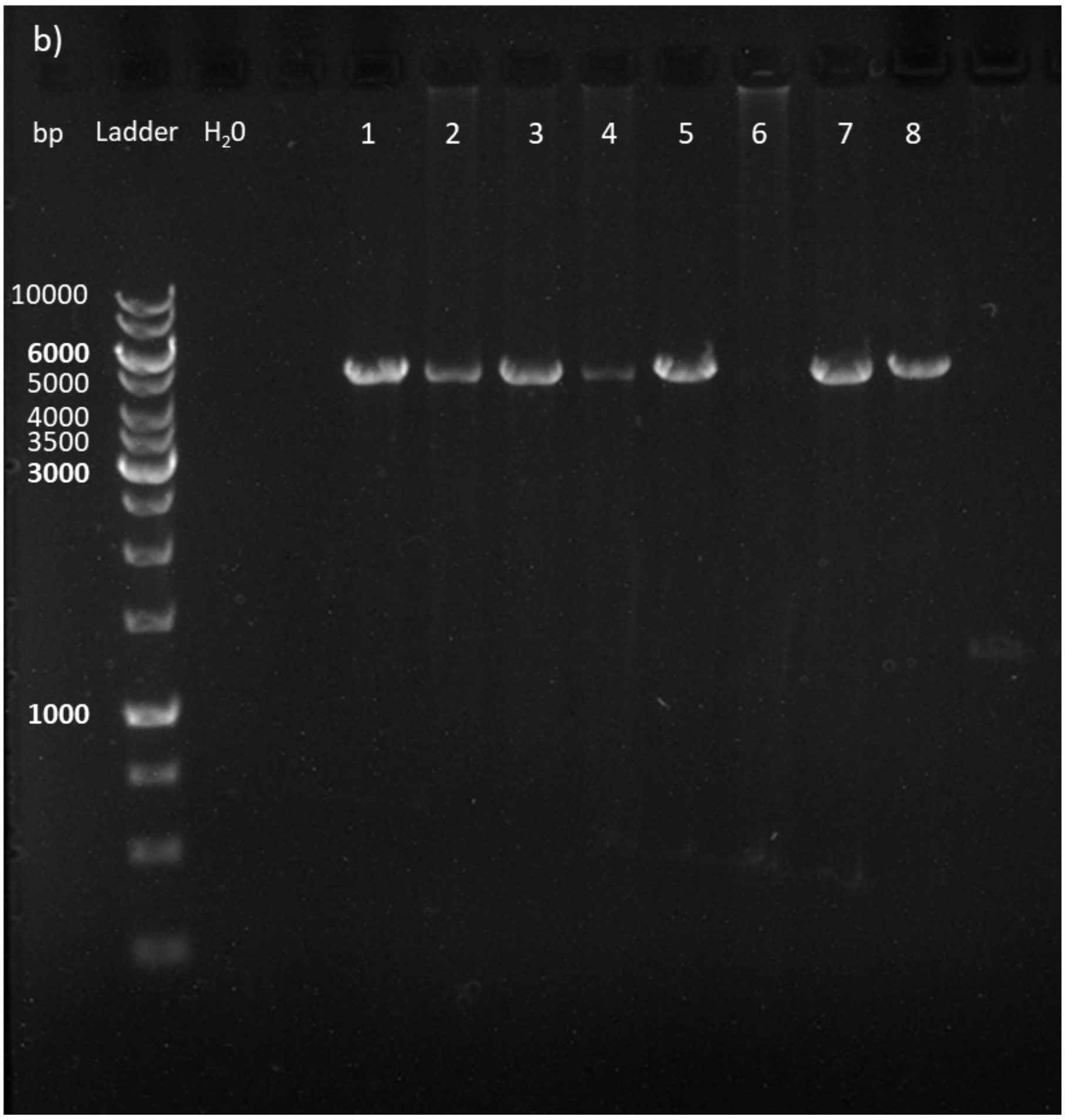
b)
bp Ladder H_20
1 2 3 4 5 6 7 8
10000
6000
5000
4000
3500
3000
1000

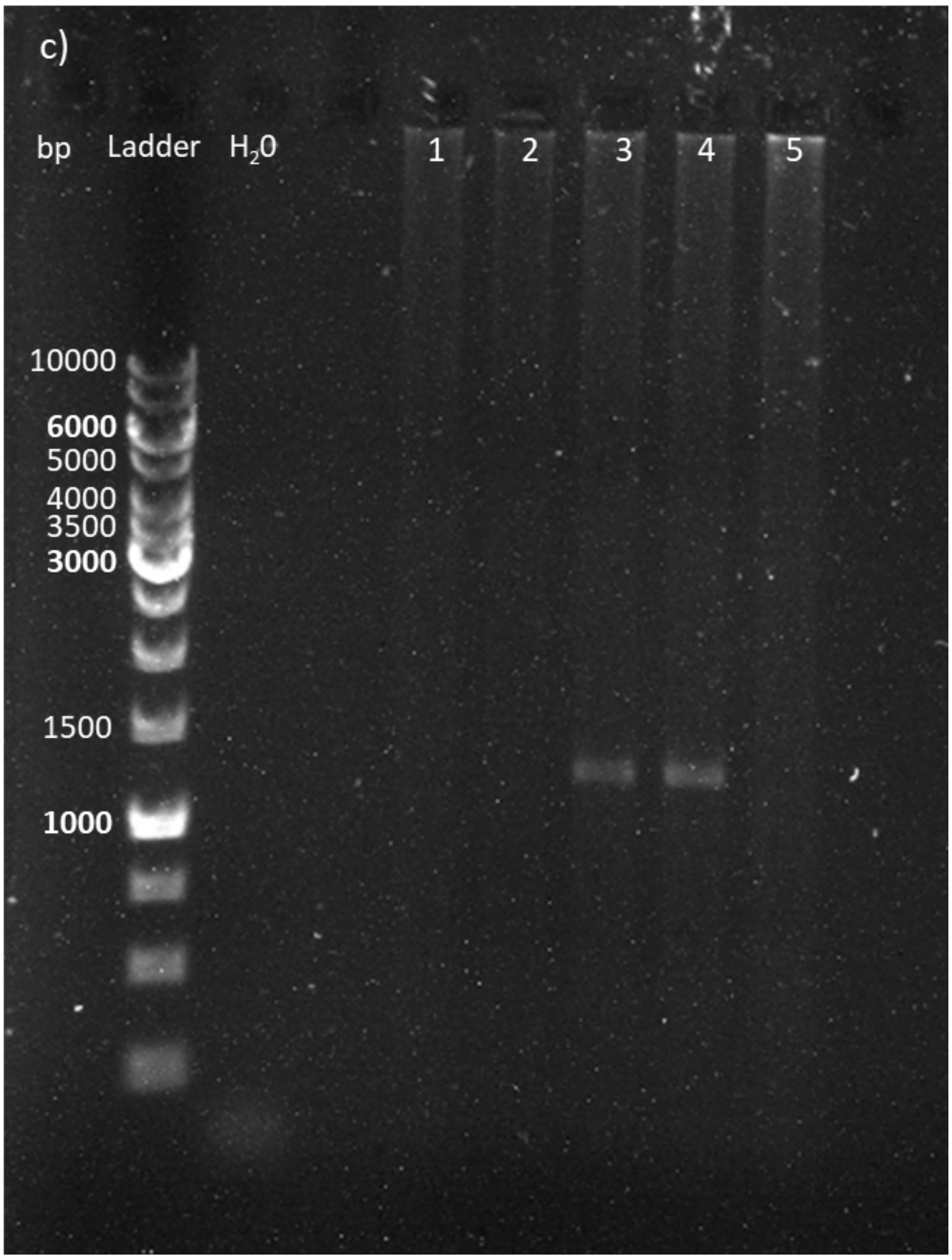
c)
bp
Ladder
H_20
1
2
3
4
5
10000
6000
5000
4000
3500
3000
1500
1000

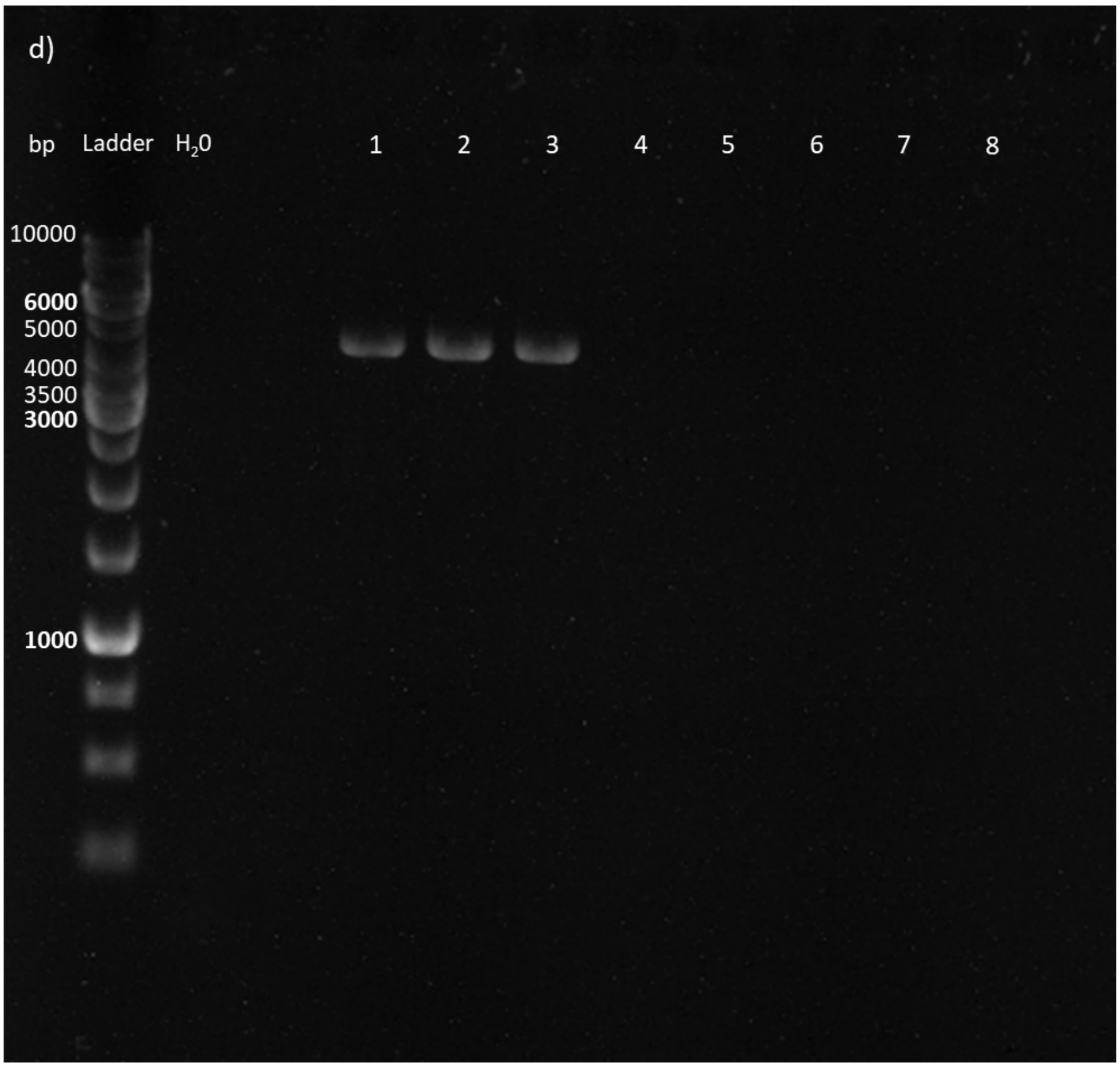
d)
bp
Ladder
H_20
1
2
3
4
5
6
7
8
10000
6000
5000
4000
3500
3000
1000

Supplementary Figure 7 Polymerase chain reaction (PCR) amplification products from eight Nordic spring oat breeding lines for three genes associated with heavy metal handling located on chromosomes 3D and 4A. Amplicons for gene a) AVESA.00010b.r2.3DG0562400 (HIPP6; 3D, 413 Mbp) with primer pair 3D_413-f/3D_413-r, b) AVESA.00010b.r2.3DG0565980 (WAK3; 3D, 430 Mbp) with primer pair 3D_430-f/3D_430-r2, c) AVESA.00010b.r2.3DG0565980 (WAK3; 3D, 430 Mbp) with primer pair 3D_430-f/3D_430-r1, and d) AVESA.00010b.r2.4AG0634750 (HIPP7; 4A, 381 Mbp) with primer pair 4A_381-f/4A_381-r.

Annotations 1 to 8 in a), b) and d) correspond to the following lines: 1 – SOA369, 2 – SOA372, 3 – Active, 4 – Hera, 5 – Welinder, 6 – SOA026, 7 – SOA599, 8 – SOA608.

Annotations 1 to 5 in c) correspond to: 1 – Active (3D_430-f/3D_430-r1), 2 – Active (3D_430-f/3D_430-r1), 3 – 140602 (3D_430-f/3D_430-r1), 4 – 140602 (3D_430-f/3D_430-r1), 5 – 140602 (3D_430-f/3D_430-r2).
